# Optimal parameter identification of solid oxide fuel cell using modified fire Hawk algorithm

**DOI:** 10.1038/s41598-024-72541-6

**Published:** 2024-09-28

**Authors:** Rahul Khajuria, Mahipal Bukya, Ravita Lamba, Rajesh Kumar

**Affiliations:** 1 Department of Electrical Engineering, Malaviya National Institute of Technology, Jaipur, India; 2 Department of Electrical and Electronics Engineering, Manipal Institute of Technology Bengaluru, Manipal Academy of Higher Education, Manipal, India; 3 Department of Hydro and Renewable Energy, Indian Institute of Technology Roorkee, Uttarakhand, India

**Keywords:** Parameter identification, Solid oxide fuel cell, Modified fire Hawk algorithm, Polarization curves, Statistical analysis, Energy science and technology, Engineering

## Abstract

An accurate and efficient approach is required to identify the unknown parameters of solid oxide fuel cell (SOFC) mathematical model for a robust design of any energy system considering SOFC. This research study proposes a modified fire hawk algorithm (MFHA) to determine the values of SOFC model parameters. The performance evaluation of MFHA is tested on two case studies. Firstly, the performance of MFHA is tested on commercially available cylindrical cell developed by Siemens at four temperatures. Results reveal that the least value of sum of squared error (SSE) is 1.04E−05, 2.30E−05, 1.03E−05, and 1.60E−05 at 1073 K, 1173 K, 1213 K, and 1273 K respectively. Results obtained using MFHA have been compared with original fire hawk algorithm (FHA) and other well established and recent algorithms. Secondly, MFHA is implemented for estimating unknown parameters of a 5 kW dynamic tabular stack of 96 cells at various pressures and temperatures. The obtained value of SSE at different temperatures of 873 K, 923 K, 973 K, 1023 K and 1073 K is 1.18E−03, 6.12E−03, 2.21E−02, 5.18E−02, and 6.00E−02, respectively whereas, SSE at different pressures of 1 atm, 2 atm, 3 atm, 4 atm, and 5 atm is 6.05E−02, 6.11E−02, 5.53E−02, 5.11E−02, and 6.64E−02 respectively.

## Introduction

Electrical energy demand is increasing globally with rapid industrialization and civilization^1^. Fossil fuels are used to fulfill this demand but their major disadvantages like global warming, exhaustible nature, carbon and harmful emissions limit their use^2^. Hence, to meet the electrical energy demand effectively, there is a need of sustainable and eco-friendly energy sources^3,4^. Currently, renewable energy resources are becoming more popular and widely adopted in meeting the electrical energy^5^. They have the advantages of being ecologically favourable, nearly no carbon emission and inexhaustible nature. In past two decades, fuel cells, being a renewable energy source, have received significant interest due to their emission free operation and high efficiency. They are now being adopted for both small and large scale power generation applications^6,7^. Different types of available fuel cells are phosphoric acid, molten carbonate, polymer exchange membrane, solid oxide, direct methanol, alkaline and reversible fuel cells^8,9^. Solid oxide fuel cell (SOFC) is regarded as the main choice because it operates at high efficiency and temperature. SOFCs are also considered as more effective because they can use natural gas directly. SOFC have the advantages like, robust nature, insensitivity to carbon monoxide, high co-generation potential, flexible with fuel input, no need of catalysts, and high efficiency (typically 60–70%) even at high temperatures^10^. All these properties of SOFC make it applicable in various applications such as transportation, waste-water treatment, distributed generation, co-generation, auxiliary power units, and space applications^11^.

A SOFC is well-known for its flexibility and quick response and manufacturers find it an emerging solution for electrical energy generation. However, the prediction of characteristics of SOFC is challenging as it involves both chemical and electrical processes. Also, to evaluate its performance under different conditions and optimize its design, an accurate SOFC mathematical model is required. The operation of a single SOFC involves many processes including electric charge transfer, heat transfer, electro-chemical reactions and mass transfer. Therefore, simultaneous occurrence of all these processes are represented by a complex mathematical model^12,13^. There are different SOFC models available in literature, like electrochemical, steady state I and steady state II model as presented in Table 1. Among these models electrochemical model is adopted in many research studies due to its effectiveness^14^ Also, the electrochemical mathematical model of SOFC contains several equations which represent various physical and chemical processes involved in its operation. However, it is a great challenge to the researchers worldwide to develop an accurate model for SOFC due to its highly non-linear nature. Nonetheless, SOFC mathematical model is complex, multi-variable and multi-modal, which make it complex and hard to control and predict its performance at different conditions. Also, it contains several unknown parameters that govern its performance and these parameters affect each other and even a small variation in these parameters greatly affects the output performance. Moreover, information about these parameters is not provided by the manufacturer. Therefore, to develop an accurate model that ensures effective online control and helps in performance assessment at different conditions such as different loads, temperatures and gas flow rates, an optimal parameter identification approach is required. Further, the behavior of SOFC is characterized by polarization characteristics which are generally a plot of current density versus power and current density versus voltage. These are obtained from the SOFC mathematical model that contains a set of voltage equations representing open circuit voltage, and different voltage drops such as concentration, ohmic, and activation voltage drop. These voltage equations, consisting of different unknown parameters, greatly affect the polarization characteristics. Therefore, a parameter identification approach is required to accurately model an SOFC in obtaining accurate polarization characteristic that matches with the experimental ones.

**Table 1:** An overview of different SOFC models and their unknown parameters.

S. no.	Model name	Mathematical modeling	Unknown parameters
1	Electrochemical model	$$\begin{aligned}&N_{cell}\left[ E_o+\frac{RT}{2F}ln\left( \frac{\left( P_{H_2} \right) ^2P_{O_2}}{\left( P_{H_2o} \right) ^2} \right) -Asinh^{-1}\left( \frac{I_{load}}{2I_{o,a}} \right) \right. \\&\left. -Asinh^{-1}\left( \frac{I_{load}}{2I_{o,c}} \right) -Bln\left( 1-\frac{I_{load}}{I_L} \right) -I_{load}R_{ohm} \right] \end{aligned}$$	$E_o, I_{oa}, A, I_{oc}, B, I_L, R_{ohm}$
2	Steady state model I	$E_o-k_E\left( T-298 \right) +\frac{RT}{4F}ln\left[ \frac{\left( P_{H_2} \right) ^2P_{O_2}}{\left( P_{H_2O} \right) ^2}\right] - \frac{2RT}{\eta _eF}$ $$\begin{aligned}&\left( sinh^{-1}\left( \frac{Iexp\left( \frac{k_2}{T} \right) }{2k_1T} \right) -ln\left( 1-\frac{k_l}{k_lln\left( 1-C_{re} \right) } \right) \right) \\ &-\left\{ \gamma exp\left[ \delta \left( \frac{1}{T_o} -\frac{1}{T} \right) \right] \right\} I\end{aligned}$$	$k_E, k_l, \gamma , \delta , k_1, k_2$
3	Steady state model II	$E_o-Asinh^{-1}\left( \frac{J}{J_o} \right) -Bln\left( 1-\frac{J}{J_{max}} \right) -IR_{ohm}$	$E_o, A, J_o, B, J_{max}, R_{ohm}$

Numerous researchers identified and estimated the parameters of SOFC using metaheuristic approaches. Guo et al.^15^ adopted modified fractional order dragonfly algorithm and minimized the SSE between empirical and obtained data. Also, results obtained were compared with other algorithms and reference work. Hao et al.^16^ proposed an adaptive chaotic grey wolf optimization (ACGWO) by improving the grey wolf optimization (GWO) algorithm using chaotic algorithm. The presented approach identified SOFC’s parameters with higher precision and convergence speed compared to other optimization algorithms in terms of accuracy, robustness, and convergence based on mean squared error (MSE) as an objective function. Bai et al.^17^ proposed a hybrid approach to identify the unknown parameter of a 5-kW physically-based dynamic tubular stack, namely cuckoo search-grey wolf optimization algorithm (CSGWO) by integrating the cuckoo search (CS) and grey wolf algorithms. The objective function was expressed as MSE and algorithm was tested at different pressures and temperatures. Additionally, results were contrasted with other well-known algorithms. Statistical results revealed that with low value of MSE, CSWO outperformed with improved precision and convergence. Wang et al.^14^ applied modified grey wolf optimization (MGWO) to extract SOFC’s parameters. In this regard, objective function to minimize the SSE was framed and the reliability of the algorithm was tested at two different scenarios based on pressure and temperature. Results revealed that MGWO proved its accuracy compared to other well-known algorithms. Kele et al.^18^ adopted a modified cat optimization algorithm to estimate SOFC’s model parameters and to minimize the error between the estimated and simulated output voltages for a 96-cell SOFC system. The results were compared with other well-established algorithms including coyote optimization algorithm (COA), chaotic binary shark smell optimization (BSSO), grass fibrous root optimization algorithm (CGFROA), and modified african vulture optimization algorithm (MAVO). Zhang et al.^19^ used an extreme learning machines (ELM) based on improved red fox optimizer (IRFO) to estimate the dynamic model parameters of SOFC. MSW was selected as the objective function and its effectiveness was evaluated and compared with other reference studies in the literature. Xiong^20^ proposed a novel competitive hybrid differential evolution and Jaya algorithm (CHDJ) by hybridizing differential evolution and Jaya algorithms to identify the model parameters of a tubular stack and cylindrical cell operating at different temperatures and pressures. MSE was taken as an objective function and effectiveness of the algorithm was validated with lower value of MSE over other well-known algorithms. Yousri et al.^21^ developed a new variant for marine predators algorithm (MPA) using a comprehensive learning and dynamic multi-swarm approach to identify the values of unknown parameters. The algorithm was named as comprehensive learning dynamic multi-swarm marine predators algorithm (CLDMMPA) and validated its performance based on steady-state and dynamic models of SOFC. Other algorithms were also adopted to provide a comparison and the effectiveness was evaluated considering non-parametric tests and statistical metrics. Ba et al.^22^ optimized the Rotor hopfield neural nertwork using GWO algorithm to estimate model parameters based on MSE-based objective function. The results revealed a good agreement with experimental ones and lesser computational difficulty. Xiong et al.^23^ proposed a simplified variant of competitive swarm optimizer (SCSO) to evaluate unknown parameters of a 5 kW dynamic tubular stack and Siemens cylindrical cell. The outcomes revealed that SCSO outperformed in terms of accuracy, convergence and robustness. Yang et al.^24^ investigated and presented a review analysis of metaheuristic approaches for identification of SOFC parameters. Wei et al.^25^ adopted chaotic binary shark smell optimizer to determine the values of steady-state and dynamic model parameters and used minimization of MSE as the objective function. Wang et al.^26^ adopted co-evolution RNA genetic algorithm (coRNA-GA) to identify values of unknown parameters of 5 kW SOFC and outcomes were contrasted considering other well-known algorithms. Hay et al.^27^ utilized interior search optimizer (ISO) to estimate transient and steady-state model parameters of SOFC based on MSE as objective function. Moreover, the flow rates of oxygen and hydrogen were also investigated and the results obtained were compared with the grasshopper optimizer, satin bowerbird algorithm and genetic algorithm. Yang et al.^28^ adopted an extreme learning machine-based method for the evaluation of 5 kW SOFC’s parameters. Shi et al.^29^ minimized SSE between the experimental and estimated voltages of SOFC model at different temperatures and pressures using converged grass fibrous root optimization algorithm (CGRA). Abaza et al.^30^ employed cyote optimizer to minimize the error between polarization characteristics and evaluated unknown parameters of SOFC.

**Table 2:** Lower and upper bounds for unknown parameters.

Parameters	$E_o ({\text {V}})$	$A ({\text {V}})$	$I_{a.e} ({\text {mA}}/{\text {cm}}^2)$	$I_{c.e} ({\text {mA}}/{\text {cm}}^2)$	$B ({\text {V}})$	$I_{lim} ({\text {mA}}/{\text {cm}}^2)$	$R_{{\text {ohm}}} ({\text {k}}\Omega \; {\text {cm}}^2)$
Lower bound (L.B)	0	0	0	0	0	0	0
Upper bound (U.B)	1.2	1	100	100	1	10,000	1

From the literature, it can be observed that many metaheuristic approaches are employed and also, many of them have been modified to obtain accurate and precise values of unknown parameters^11,18,31,32^. However, due to non-linearity and complexity of SOFC model, there is still a scope to check the reliability of recently developed metaheuristic algorithms^33,34^. Moreover, above mentioned approaches still face challenges such as poor convergence, dependency on controlling parameters, stuck in local minima, high computational time, lesser exploration and exploitation over iterations^35^. Over the past two decades, many recent optimization algorithms inspired by natural phenomena have been developed^36,37^ such as Puma optimizer^38^, levy flight arithmetic algorithm^39^, footballtraing algorithm^40^, hiking optimization algorithm^41^. Additionally, metaheuristic algorithms are also improved for parameter estimation of solid oxide fuel cell such as improved version of cat and mouse optimization^42^, repairable grey wolf optimizer^43^, co-operation search algorithm^44^ etc. However, they solve non-linear and complex optimization problems depending on their own ability. Furthermore, No Free Lunch (NFL) theorem states that no algorithm can provide an equally effective and accurate solution to every optimisation problem^7^. In recent time, many effective and efficient metaheuristic algorithms have been proposed and applied in solving complex and non-linear optimization problems. Furthermore, SOFC parameter identification problem can also be framed as an optimization problem and solved using metaheuristic algorithms.

Recently, Fire hawk optimizer (FHO) was proposed by Azizi et al.^45^ that mimics the behavior of brown falcons and black kites in catching the prey. Fire hawks are responsible for spreading fire in nature and perform destructive phenomena intentionally using fire sticks by carrying them in their talons. They perform this action to control and capture the prey and release burning sticks in the unburnt locations where large number of preys exist to complete their task. This destructive phenomenon forced these preys to escape from safe zones in a panicked manner and helpful in attack of fire hawks to catch them easily. Thus, the fire hawk optimizer simulated the destructive phenomenon of spreading the fire and catching the prey. Since, FHO is a recent metaheuristic algorithm and its performance was investigated on two hundred thirty three test functions with 2–100 dimensions. Also, a fair comparison was conducted among ten traditional and new optimization algorithms which further proves its superiority over other algorithms in solving different problems^46^. However, FHA works differently from other developed optimization algorithms such as such as the Whale Optimization Algorithm (WOA) and Particle Swarm Optimization (PSO). FHA fundamentally differs from these approaches, in terms of its inspiration, exploration and exploitation mechanisms, and overall structure. FHA is based on the unique behavior of fire hawks, which intentionally spread wildfires to flush out prey. This natural behavior forms the foundation of how the algorithm navigates the search space. FHA models the movement of hawks toward prey (optimal solutions) by strategically spreading fire (exploring the search space) and concentrating on areas with higher prey density (exploiting good solutions). The exploration phase in FHA involves the spread of fire across the search space, representing the scattering of potential solutions. Hawks then focus on regions with higher prey density, refining their search in those areas. This dual strategy allows FHA to dynamically adjust its focus between exploration (searching new areas) and exploitation (refining good solutions) based on the prey density in different regions of the search space.

On the other hand, WOA is inspired by the bubble-net feeding strategy of humpback whales. The algorithm mimics the spiral and encircling behaviors of whales as they hunt, translating these behaviors into mechanisms for exploration and exploitation within the search space. WOA alternates between two main phases: encircling prey (exploitation) and spiral updating (exploration). The algorithm can switch between these modes based on a probability factor, enabling it to balance the search between local and global exploration. Whales encircle prey and then create a spiral to attack, simulating a convergence process toward the optimal solution. PSO, in contrast, is inspired by the social behavior of bird flocks or fish schools. Each particle in the swarm, representing a potential solution, adjusts its position based on its own experience (personal best) and the experience of neighboring particles (global best). The movement of particles simulates the social sharing of information, guiding the swarm toward the best solutions found so far. PSO’s exploration is driven by the random initial positions and velocities of particles, while exploitation is guided by the best solutions identified by the swarm. The balance between exploration and exploitation in PSO is controlled by the inertia, cognitive, and social components within the velocity update equation.

Recently FHA is applied in different fields of engineering such as Hosseinzadeh et al. proposed a cluster-based trusted routing method using fire hawk optimizer in wireless sensor networks^47^. Mudhsh et al. applied machine learning approaches to model thermo-hydraulic behavior of a helical heat exchanger and fire hawk optimizer was used to optimize a random vector functional link as a powerful machine learning approach^48^. Jasmine et al. proposed a hybrid techniques which combines spiking neural network and fire hawk algorithm for EV parking lot and optimal location of capacitors in voltage and power loss^49^. Kumari et al. utilized FHA for dynamic load balancing in cloud environment to enhance to enhance the performance of system considering multi attributes^50^. Mehadi et al. applied dynamic clustering scheme based on fire hawk optimizer for flying ad hoc networks to determine the role of each UAV cluster head or cluster member in the network to maintain network performance and increases in scalability^51^. However, the slow convergence and local minima trap are two challenges in solving any optimization problem which are needed to be solved by modifying the algorithm.

In summary, it is concluded form the above discussions that the mathematical modeling of SOFCs plays a crucial role in simulating and predicting their behavior within energy systems and other operational conditions. This model is essential for simulating SOFC performance under various operating conditions and optimizing their operation in applications like fuel cell integration with renewable energy sources, energy management, energy storage systems, and microgrids. Furthermore, these models are helpful in monitoring SOFC performance, ensuring efficient control, and maximizing the effective use of the system. Therefore, to accurately replicate real-time SOFC operation, it is necessary to precisely identify seven unknown parameters within the model. This precise parameter estimation is vital for effective modeling, control, and optimization of SOFCs. Although many metaheuristic algorithms have been employed for parameter estimation in SOFCs as mentioned in literature, they often encounter challenges related to the balance between exploration and exploitation. These algorithms may require extensive tuning, struggle with convergence speed, and risk becoming trapped in local minima. Therefore, in this paper modified fire hawk algorithm (MFHA) is proposed in identifying unknown parameters of SOFC and objective function is defined as minimization of the sum of squared error. The main aim is to develop a parameter identification approach for SOFC and to minimize the error function considering all the constraints. MFHA evaluates seven unknown parameters and is proposed as a good identifier, considering a close mapping of experimental and estimated polarization characteristics. To demonstrate the superiority of MFHA, a comparison analysis with well-known algorithms is conducted. The robustness of algorithm is tested at two different case studies. Moreover, statistical study, non-parametric test, box plot, and convergence curve analysis have been assessed to demonstrate the reliability and robustness of the MFHA in identifying seven parameters of SOFC mathematical model. The principle contributions of this study are illustrated as follows:

- Proposing a modified fire hawk algorithm with improved accuracy for identification of SOFC model parameters.
- Implementing MFHA on benchmark functions and comparing results with original fire hawk algorithm.
- Evaluating the effectiveness of MFHA in estimating unknown model parameters of SOFC.
- Comparing MFHA with well-known metaheuristic optimization algorithms and already established work.
- Identifying the accuracy of algorithm considering closeness between the estimated and experimental polarization characteristics (I–V and P–V curves).
- Validating reliability and robustness of MFHA at two different case studies.
- Performing statistical study, convergence analysis, box plot analysis, and non-parametric test to demonstrate the superiority of MFHA over other algorithms.

The rest of the article is organized as follows. “Mathematical modeling of SOFC” describes the mathematical modeling of SOFC and problem formulation. In “Fire Hawk algorithm”, fire hawk algorithm and steps for modification have been described. Results and discussions are provided in “Results and discussions”. Finally, the conclusion is given in “Conclusions”.

**Table 3:** Benchmark functions.

Test cases	Dimensions	Range	${f_{min}}$
$F_{01}(x)=\sum _{i=1}^{n}x_{i}^2$	50	[− 100 to 100 ]	0
$F_{02}(x)=\sum _{i=1}^{n}\left| x_{i}\right| +\prod _{i=1}^{n}\left| x_{i}\right|$	50	[− 100 to 100 ]	0
$F_{03}(x)=\sum _{i=1}^{n}\left( \sum _{j-1}^{n} \right) x_{j}$	50	[− 100 to 100 ]	0
$F_{04}(x)=Max_{i}\left\{ {\left| x_{i} \right| ,1\le i\le n }\right\}$	50	[− 100 to 100 ]	0
$F_{05}(x)=\sum _{i=1}^{n-1}\left[ 100(x_{i+1})^2 +(x_{i}-1)^2 \right]$	50	[− 30 to 30 ]	0
$F_{06}(x)=\sum _{i=1}^{n}(\left[ x_{i} + 0.5\right] )^2$	50	[− 100 to 100 ]	0
$F_{07}(x)=\sum _{i=1}^{n}ix_{i}^4 + random[0,1)$	50	[− 1.28 to 1.28 ]	0
$F_{08}(x)=\sum _{i=1}^n -x_isin\left( \sqrt{\left| x_{i} \right| } \right)$	50	[− 500 to 500 ]	-418.9829*5
$F_{09}(x)=\left[ x_i^2-10cos(2\pi x_{i}) + 10 \right]$	50	[− 5.12 to 5.12 ]	0
$F_{10}(x)= -20 exp(-0.2 \sqrt{\frac{1}{n} \sum _{i=1}^n x_i^2}) - exp(\frac{1}{n} \sum _{i=1}^n cos(2\pi x_i)) + 20 + e$	50	[− 32 to 32 ]	0
$F_{11}(x)=1/400\sum _{i=1}^n x_i^2-\prod _{i=1}^n cos(x_i/\sqrt{i})+1$	50	[− 600, 600]	0
$F_{12}(x)=\pi /n\left\{ 10sin(\pi y_{i})+\sum _{i=1}^{n-1}(y_{i}[1+10sin^2(\pi y_{i+1})]+(y_n-1)^2 \right\} + \sum _i^nu(x_i,10,100,4)$ $$y_i=1+\frac{x_i+1}{4}, u(x_i,q,k,m)=\left\{ \begin{matrix} k(x_i-a)^m & x_i>a\\ o& -a<x_i<a \\ k(-x_i-a)^m& x_i<a \end{matrix}\right. $$	50	[− 50, 50]	0
$F_{13}(x)=0.1\left\{ sin^2(3\pi x_i)+\sum _{i=1}^n (x_i -1)^2\left[ 1+ sin^2(3\pi x_i+1) \right] +(x_n-1)^2\left[ 1+sin^2(2\pi x_n) \right] \right\}$ $\sum _{i=1}^n u(x_i,5,100,4)$	50	[− 50, 50]	0
$F_{14}(x)=\left( \frac{1}{500}+\sum _{j=1}^{25}\frac{1}{j+\sum _{i=1}^{2}(x_i-a_{ij})^6}\right) ^{-1}$	2	[− 65.536, 65.536]	1
$F_{15}(x)=\sum _{i=1}^{11}\left[ a_i-\frac{x_1(b_i^2+b_ix_2)}{b_i^2+b_ix_3+x_4}\right] ^2$	4	[− 5, 5]	0.00030
$F_{16}(x)=(4x_1^2-2.1x_1^4+\frac{1}{3}x_1^6+x_1x_2-4x_2^2+4x_2^4)$	2	[− 5, 5]	-1.0316
$F_{17}(x)=(x_2-\frac{5.1}{4\pi ^2}x_1^2+\frac{5}{\pi }x_1-6)^2+10(1-\frac{1}{8\pi })\cos x_1+10$	2	[− 5, 5]	0.398
$F_{18}(x)=\left[ 1+(x_1+x_2+1)^2(19-14x_1+3x_1^2-14x_2+6x_1x_2+3x_2^2) \right]$ $\times \left[ 30+(2x_1-3x_2)^2\times (18-32x_1+12x_1^2+48x_2-36x^1x^2+27x_2^2)\right]$	2	[− 5, 5]	3
$F_{19}(x)=-\sum _{i=1}^{4}c_iexp(-\sum _{j=1}^{3}a_{ij}(x_j-p_{ij})^2$	3	[0, 1]	-3.86
$F_{20}(x)=-\sum _{i=1}^{4}c_iexp(-\sum _{j=1}^{6}a_{ij}(x_j-p_{ij})^2)$	6	[0, 1]	-3.32
$F_{21}(x)=-\sum _{i=1}^{5}\left[ (X-a_i)(X-a_i)^T+c_i \right] ^{-1}$	4	[0, 10]	-10.1532
$F_{22}(x)=-\sum _{i=1}^{7}\left[ (X-a_i)(X-a_i)^T+c_i \right] ^{-1}$	4	[0, 10]	-10.4028
$F_{23}(x)=-\sum _{i=1}^{10}\left[ (X-a_i)(X-a_i)^T+c_i \right] ^{-1}$	4	[0, 10]	-10.5363

## Mathematical modeling of SOFC

The electrochemical model is used in obtaining the polarization characteristics of SOFC. This model describes the output voltage and consists of different voltage equations that are used to model the behavior of the SOFC. The SOFC stack output voltage can be expressed as follows^16^:

1 $$\begin{aligned} E_{stack}=N_c*E_{cell}, \end{aligned}$$

where, $E_{stack}$ represents stack output voltage and $E_{cell}$ represents the single cell output voltage. $N_{c}$ represents the total number of series connected cells. A single SOFC’s output voltage is represented as follows^16^:

2 $$\begin{aligned} E_{cell}= E_{o.c}-E_{act.}-E_{conc.}-E_{ohm.}, \end{aligned}$$

where, $E_{o.c}$ represents open circuit voltage, $E_{act.}$, $E_{conc.}$ and $E_{ohm.}$ represent activation, concentration and ohmic voltage losses respectively. The open circuit voltage is expressed as follows^16^:

3 $$\begin{aligned} E_{o.c}= E_o+\frac{R*T}{4F}ln\left[ \frac{{P_{H_{2}}}^{2}P_{O_{2}}}{P_{H_{2}O}^{2}} \right] , \end{aligned}$$

where, $E_{o}$ is reversible voltage, *T*, $F(=96,486 C/mol)$, and $R (= 8.314 kJ/kmol.K)$ represent operating temperature, Faraday’s constant and Gas constant respectively. $P_{H_{2}O}$, $P_{O_{2}}$, and $P_{H_{2}}$ represent saturated pressure of water, oxygen, and hydrogen respectively. Activation, concentration and ohmic voltages for a SOFC can be expressed as follows^16^:

4 $$\begin{aligned} E_{act.}=A\left( \sinh ^{-1}\left( \frac{I_{l}}{2I_{a.e}} \right) + \sinh ^{-1}\left( \frac{I_{l}}{2I_{c.e}} \right) \right) , \end{aligned}$$

where, $I_{l}$ represents the load current density (mA/cm$^2$). $I_{c.e}$ and $I_{a.e}$ represent cathode and anode exchange current density (mA/cm$^2$) respectively. *A* represents the Tafel slope in (V). The concentration voltage loss is given as follows:

5 $$\begin{aligned} E_{conc.}=Bln\left( 1-\frac{I_{l}}{I_{lim}} \right) , \end{aligned}$$

where, B represents a coefficient in Volt and $I_{lim}$ represents the limiting current density (mA/cm$^2$). The ohmic voltage loss is given by^16^:

6 $$\begin{aligned} E_{ohm.}=I_{l}R_{ohm}, \end{aligned}$$

where $R_{ohm}$ represents the ohmic resistance (k$\Omega$ cm$^2$). Based on the given equations, there are seven parameters including $E_{o}$, *A*, $I_{a.e}$, $I_{c.e}$, *B*, $I_{lim}$, and $R_{ohm}$, whose values are unknown and need to be identified optimally. The process of parameter identification can be converted into an optimization problem by describing an objective function subject to constraints and can be solved by optimization algorithms.

### Parameter identification of SOFC model

The major limitations in developing an accurate SOFC model are (i) there is no information of several parameters that are used in mathematical model on fuel cell data sheets (ii) any small variation in the values of these parameters greatly influence the polarization characteristics and (iii) any inaccurate value of these parameters can largely deviate the simulated polarization characteristics from actual one. Therefore, to build an accurate SOFC model considering equations (1)–(6), the values of parameters like $E_{o}$, *A*, $I_{a.e}$, $I_{c.e}$, *B*, $I_{lim}$, and $R_{ohm}$, should be accurately estimated. Also, the parameter extraction process for SOFC is considered as a highly non-linear, multivariate and challenging task. Therefore, SOFC model parameter identification problem can be solved by defining it as an optimization problem. The objective function for this problem is framed as minimization of the sum of squared error that is described as follows^14,29^:

7 $$\begin{aligned} F_{obj} = min \left( SSE= \sum _{j=1}^{K}\left( E_{j}^{exp}(I_l)-E_{j}^{est}(I_l,X_i) \right) ^{2}\right) . \end{aligned}$$

Subject to:

8 $$\begin{aligned} I_{c.e}< I_{a.e}, \end{aligned}$$

9 $$\begin{aligned} I_{l}< I_{lim}, \end{aligned}$$

10 $$\begin{aligned} X_{L.B}<X_i< X_{U.B}, \end{aligned}$$

where, *K* represents the number of experimental data points. $$E_{j}^{exp}$$ denotes experimental voltage values corresponding to given current density $$I_l$$, and $$E_{j}^{est}(I_l,X_i)$$ denotes the estimated voltage values by using optimization algorithm corresponding to given $$I_l$$ current density values and $$X_i$$ represents seven unknown parameters. $$X_{L.B}$$ and $$ X_{U.B}$$ represent lower and upper limits for unknown parameters and are tabulated in Table 2 The main task is to minimize the sum of squared error between experimental and estimated voltages subject to given constraints and identify the optimal value of seven unknown parameters.

**Table 4:** Parameter setting of different algorithms.

BES^52^	PSO^53^	DE^54^	GWO^55^	WOA^56^	MRFO^57^	AEO^58^	HBA^59^	GWO-WOA^60^
a = 10	$C_1 = 2$	Weighting factor = 0.1	a = Linearly decreasing from 2 to 0	a = Linearly decreasing from 2 to 0	$\beta = 1.5$	PR = 0.3	$r_{1}, r_{2}$ = Random number [0,1]	a = Linearly decreasing from 2 to 0
R = 1.5	$C_2 = 2$	Crossover rate = 0.9	A = [− a, a]	A = [−a, a]	$r_1,r_2$ = Random number [0,1]	CR = 0.5	$F = \{-1,1\}$	A = [−a, a]
$\alpha = 2$	w = 0.4	Strategy = 1	C = Random number [0,1]	C = Random number [0, 1]	S = 2	DR = 0.4	p = 0.5	C = Random number [0, 1]
$C_1 = 2$	Number of iterations = 1000	Number of iterations = 1000	Number of iterations =1000	b = 1	Number of iterations = 1000	$\alpha = 1$	$\beta = 6$	b = 1
$C_2=1$	Population size = 30	Population size = 30	Population size = 3	l = [− 1,1]	Population size = 30	Number of iterations = 1000	Number of iterations = 1000	l = [− 1,1]
Number of iterations = 1000				Number of iterations = 1000		Population size = 30	Population size = 30	p = 0.6
Population size = 30				Population size = 30				Number of iterations = 1000
								Population size = 30

## Fire Hawk algorithm

Fire hawk algorithm (FHA) is a recently developed metaheuristic algorithm that emulates the foraging nature of brown falcons, whistling and black kites^45^ and can be seen in nature to perform the destructive phenomenon of spreading fire. They intentionally spread fire in nature to capture and control the prey with the help of burning sticks. They carry burning sticks in their talons and beaks to perform this action. The prey includes snakes, rodents and other animals. Fire hawk birds collect burning sticks and release them to set the fire in unburned locations where large numbers of prey exist. These preys are horrified by these small fires and are forced to escape in a panicked and hustled manner. This terrifying phenomenon makes it easier for fire hawks to capture their prey^45^.

### Mathematical formulation of fire Hawk behavior

**Fig. 1:**
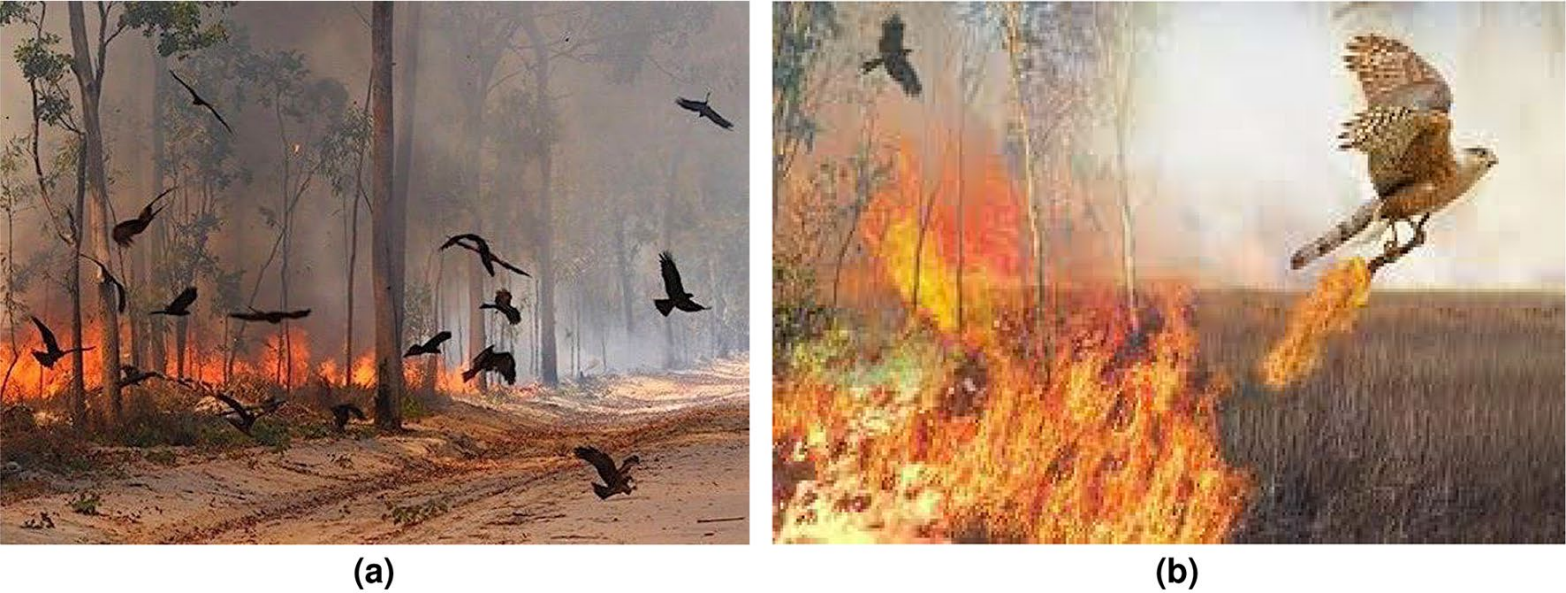
Behavior of fire Hawk to spread fire^45^.

Fire hawk algorithm imitates the behavior of fire hawks in setting and spreading fire to catch the prey. The behavior of fire hawk around fires can be represented in Fig. 1. Mathematically, the behavior of fire hawks can be formulated in a detailed manner using the following steps. Firstly, population candidates in search space are given as follows:

11 $$\begin{aligned} P=\begin{bmatrix} P_{1}\\ P_{2}\\ \vdots \\ P_{i} \\ \vdots \\ P_{N} \end{bmatrix}=\begin{bmatrix} p_{1}^{1}&  p_{1}^{2} &  \cdots &  p_{1}^{j} &  \cdots & p_{1}^{d} \\ p_{2}^{1}&  p_{2}^{2} &  \cdots &  p_{i}^{j} &  \cdots & p_{i}^{d} \\ \cdots & \cdots &  \cdots & \cdots &  \cdots & \cdots \\ p_{N}^{1}&  p_{N}^{2} &  \cdots &  p_{N}^{j} &  \cdots & p_{N}^{d} \\ \cdots & \cdots &  \cdots & \cdots &  \cdots & \cdots \\ p_{N}^{1}&  p_{1}^{2} &  \cdots &  p_{1}^{j} &  \cdots & p_{1}^{d} \\ \end{bmatrix} , \left\{ \begin{matrix} i=1,2, \ldots ,n\\ j=1,2,\ldots ,d \end{matrix}\right. \end{aligned}$$

where, $P_{i}$ is the $i_{th}$ solution candidate in the search space, *d* indicates number of dimensions , *N* represents the total solution candidate number. To determine the position of fire hawks and preys, a random initialization is used as follows^45^:

12 $$\begin{aligned} p_{i}^{j}=p_{i,L.b}^{j}+rand.(p_{i,U.b}^{j}-p_{i,L.b}^{j}), \left\{ \begin{matrix} i=1,2,....,n\\ j=1,2,...,d \end{matrix}\right. \end{aligned}$$

where, $$ p_{i}^{j}$$ represents decision variable. $$p_{i,L.b}^{j}$$ and $$p_{i,L.b}^{j}$$ are lower and upper limits for the $$j_{th}$$ decision variable. *rand* represents a random number between 0 and 1.

**Fig. 2:**
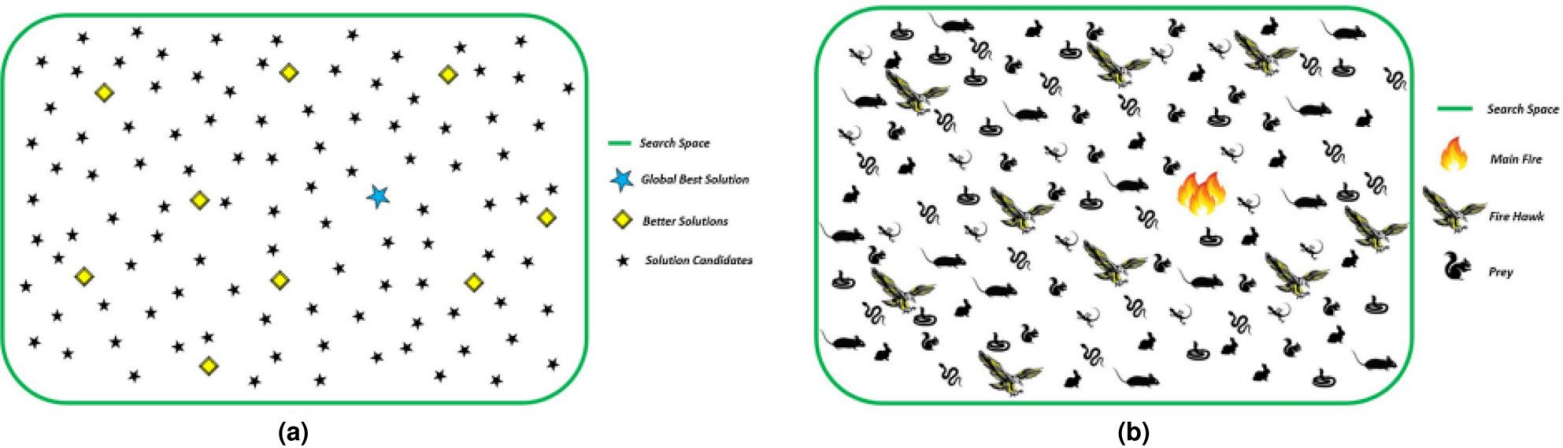
Schematic representation of determining the position of prey and fire hawk in search space^45^.

**Fig. 3:**
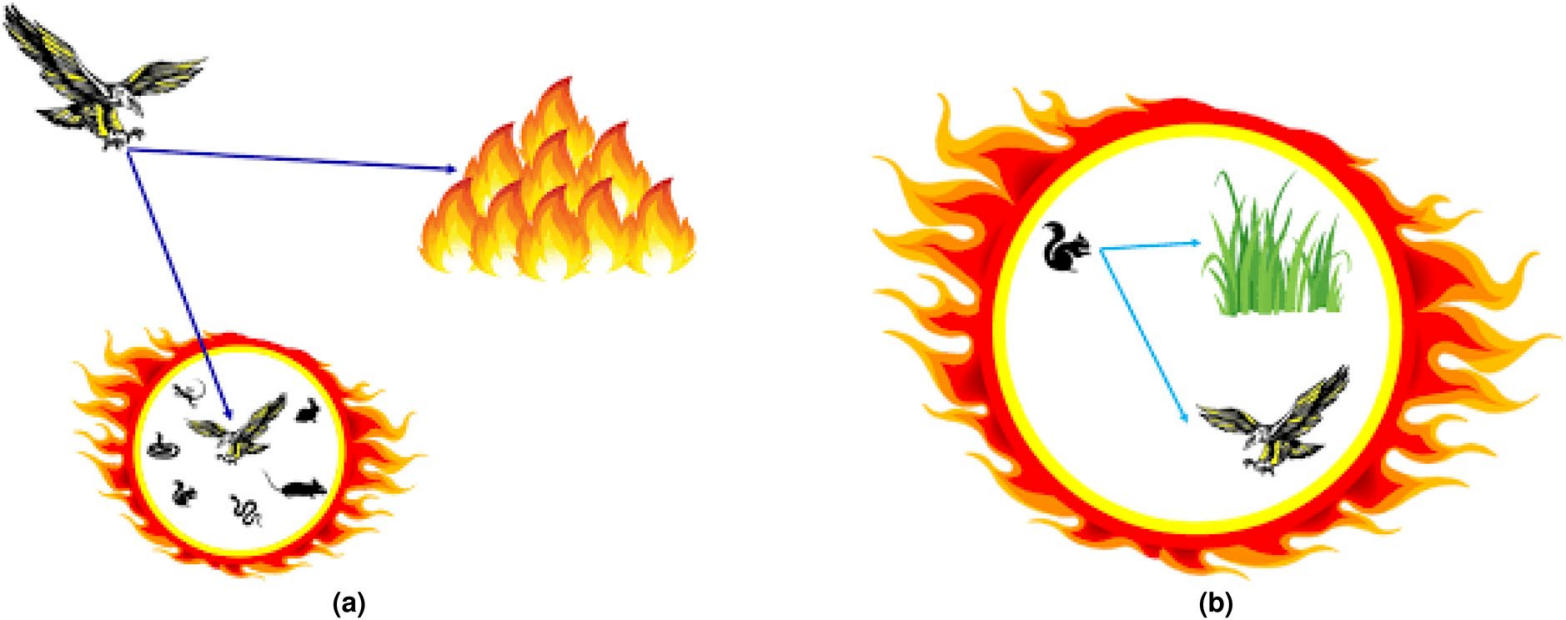
Schematic representation of (**a**) updating position of fire hawk in the search space, (**b**) updating position of prey inside fire hawk’s territory^45^.

Step 1: Location of prey and Fire hawk: The location of fire hawk and prey can be selected based on the evaluation of the objective function. Solution candidates having superior objective function evaluations are considered as fire hawks and that with poor evaluations are considered as prey.

Step 2: Spreading the fire: To spread fire around the prey and make the foraging easier, the selected fire hawks are used. The first fire used by the fire hawks is represented as global best solution. The schematic representation of this behavior can be represented as shown in Fig. 2a,b and the corresponding mathematical formulation can be given as follows^45^:

13 $$\begin{aligned} PR=\begin{bmatrix} PR_{1}\\ PR_{2}\\ \vdots \\ PR_{k}\\ \\ PR_{m} \end{bmatrix},\left\{ \begin{matrix} k=1,2,\ldots ,m \end{matrix}\right. \end{aligned}$$

14 $$\begin{aligned} FH=\begin{bmatrix} FH_{1}\\ FH_{2}\\ \vdots \\ FH_{l}\\ \vdots \\ FH_{n} \end{bmatrix},\left\{ \begin{matrix} l=1,2,\ldots ,n \end{matrix}\right. \end{aligned}$$

where, $PR_k$ is the $k_{th}$ prey among *m* preys. $FH_l$ represents $l_{th}$ fire hawk in the search space having *n* fire hawks.

Step 3: Calculation of distance between fire hawk and prey: To determine the effective territory, the distance between fire hawk and prey can be evaluated to locate the nearest prey. The illustration of this behavior can be represented in Fig. 3a,b and can be calculated as follows^45^:

15 $$\begin{aligned} D_{k}^{l}=\sqrt{\left( a_{2}- a_{1}\right) ^{2}+\left( b_{2}- b_{1}\right) ^{2}}, \left\{ \begin{matrix} l=1,2,\ldots ,n.\\ k=1,2,\ldots ,m. \end{matrix}\right. \end{aligned}$$

where, $D_{k}^{l}$ represents the total distance between $k_{th}$ prey and $l_{th}$ fire hawk. $( a_{2}- a_{1})$ and $( b_{2}- b_{1})$ denotes the coordinates of fire hawk and prey, respectively.

After calculating the distance, the territory of each of the fire hawks is differentiated as per the nearest prey around them. Fire hawk determines their specific territory by using the criteria as the fire hawk with superior evaluation chooses the best nearest prey and that with poor evaluation selects the next nearest prey.

Step 4: Updating the position: In this step, fire hawk sets the fire by using the collected fire sticks from the main fire. Each fire hawk drops the collected burning sticks in a specific territory. This process forces prey to fly hastily. Simultaneously, some of the birds may utilize burning sticks from the territories of other fire hawks. These two behaviors in updating the positions can be used and mathematically represented as follows^45^:

16 $$\begin{aligned} FH_{l}^{new}= FH_{l}+\left( r_{1}\times G.B-r_{2}\times FH_{near} \right) , l=1,2,\ldots ,n \end{aligned}$$

where, $FH_{l}^{new}$ represents the new position, *G*.*B* represents the global best position. $FH_{near}$ represents the one of the other fire hawk present in the search space. $r_{1}$ and $r_{2}$ represent the uniformly distributed random numbers between 0 and 1. The position is also updated when a fire hawk releases a fire stick towards the prey. In this process, the prey decides to hide or run hastily in the direction of fire hawk accidentally. This is simulated as follows^45^:

17 $$\begin{aligned} PR_{q}^{new}=PR_{q}+\left( r_{3}\times FH_{l}-r_{4}\times SP_{l} \right) ,\left\{ \begin{matrix} l=1,2,\ldots ,n\\ q =1,2,\ldots ,r \end{matrix}\right. \end{aligned}$$

where, $PR_{q}^{new}$ represents $q_{th}$ prey’s new location nearby $l_{th}$ fire hawk, $SP_{l}$ represents the safe place under $l_{th}$ fire hawk territory. $r_3$ and $r_4$ are random numbers (0, 1).

Apart from this, the prey may move in the direction of the other fire hawk’s territory and there is a possibility that the prey may move more closely towards fire hawks or the prey tries to prevent from being seen and move towards safe place outside the territory of fire hawk. These actions can be used to update the position and can be mathematically formulated as follows^45^:

18 $$\begin{aligned} PR_{q}^{new}=PR_{q}+\left( r_{5}\times FH_{Alter}-r_{6}\times SP \right) ,\left\{ \begin{matrix} l=1,2,\ldots ,n\\ q =1,2,\ldots ,r \end{matrix}\right. \end{aligned}$$

*SP* denotes a safe place presents at the outside of territory of $l_{th}$ fire hawk. $r_5$ and $r_6$ represent random numbers.

**Fig. 4:**
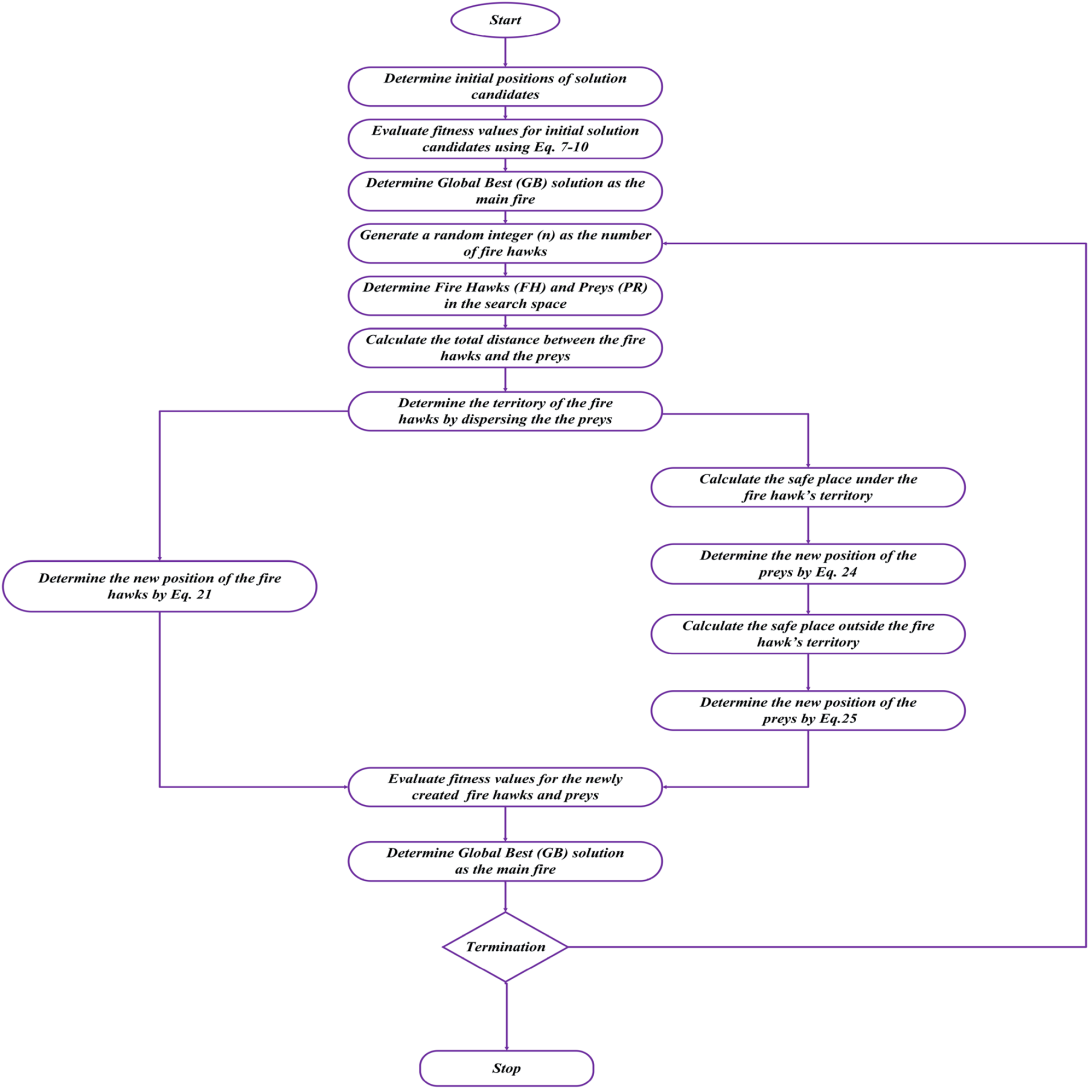
Flowchart of the modified fire hawk algorithm.

Safe place in nature can be considered as a place where most of the animals exist together to remain safe during any danger or hazard. Mathematically, safe place inside and outside the fire hawk’s territory can be represented as follows^45^:

19 $$\begin{aligned}  &   SP_{l}=\frac{\sum _{q=1}^{r}PR_{q}}{r}, \left\{ \begin{matrix} q=1,2,\ldots ,r\\ l=1,2,\ldots ,n \end{matrix}\right. \end{aligned}$$

20 $$\begin{aligned}  &   SP=\frac{\sum _{k=1}^{m}PR_{k}}{m}, k=1,2,\ldots ,m \end{aligned}$$

where, $PR_{q}$ represents $q_{th}$ prey surrounded by $l_{th}$ fire hawk in the search space.

### Modified fire Hawk algorithm (MFHA)

A recently developed fire hawk algorithm offers various advantages in addressing different benchmark problems. However, in some of the cases, it may converge prematurely and stuck in local optima. To overcome this issue, the algorithm needs to be modified as per the level of the problem. Recently, many improvements have been adopted in existing algorithms to make them fast and produce accurate results. For better convergence and to avoid the local optima trap, the improvements have been adopted in step 4 for updating the position. In step 4, the first phase is to force the prey to fly hastily and it is achieved when fire hawks drop the burning sticks in their specific territory. This phase is modified as follows:

21 $$\begin{aligned} FH_{l}^{new}= FH^{l}+\left( Ir_{1}\times G.B-Ir_{2}\times FH_{near} \right) , l=1,2,\ldots ,n \end{aligned}$$

where, $Ir_1$ is a factor of improvement which is a linearly decreasing factor varying from 1 to 0 as the iterations progress. $Ir_2$ is another factor of improvement that is adopted in the algorithm. These factors are described as follows:

22 $$\begin{aligned} Ir_1= 1-\frac{t}{t_{max}} \end{aligned}$$

where, *t* is the iteration in progress and $t_{max}$ denotes the total number of iterations.

23 $$\begin{aligned} Ir_2=\alpha _{min}+\left( \alpha _{max}-\alpha _{min} \right) *p^{\left( K-1 \right) } \end{aligned}$$

where, $\alpha _{min} = 0.1$, $\alpha _{max}=0.9$, $p=0.95$, and K denotes a random number (0,1).

The second phase in step 4 is the determination of the movement of preys towards safe places inside and outside of the territory of each fire hawk. These phases are modified as follows:

24 $$\begin{aligned}  &   PR_{q}^{new}=PR_{q}+\left( Ir_{1}\times FH_{l}-Ir_{2}\times SP_{l} \right) ,\left\{ \begin{matrix} l=1,2,\ldots ,n\\ q =1,2,\ldots ,r \end{matrix}\right. \end{aligned}$$

25 $$\begin{aligned}  &   PR_{q}^{new}=PR_{q}+\left( r\times FH_{Alter}-Ir_{2}\times SP \right) ,\left\{ \begin{matrix} l=1,2,\ldots ,n\\ q =1,2,\ldots ,r \end{matrix}\right. \end{aligned}$$

**Table 5:** Results obtained for benchmark functions using FHA and MFHA.

Function	FHA				MFHA			
	Min	Max	Mean	Standard deviation	Min	Max	Mean	Standard deviation
$F_{01}$	9.9E−175	6.2E−160	2.1E−161	0	0	3.5E−240	1.2E−241	0
$F_{02}$	1.87E−46	1.51E−41	1.8E−42	3.83E−42	3.19E−74	6.23E−68	5.95E−69	1.59E−68
$F_{03}$	7.6E−178	3.6E−159	1.6E−160	0	4.9E−271	5E−242	1.7E−243	0
$F_{04}$	4.9E−70	1.59E−63	1.12E−64	3.45E−64	2.52E−79	5.08E−72	4.36E−73	1.05E−72
$F_{05}$	0.024908	0.629076	0.193571	0.155339	0.012881	0.089163	0.124964	0.028001
$F_{06}$	0.000401	0.008758	0.00399	0.001754	0.00733	0.197013	0.028658	0.034316
$F_{07}$	1.1E−05	0.00185	0.000578	0.000425	1.01E−05	0.00074	0.000333	0.000202
$F_{08}$	− 4189.83	− 4189.12	− 4189.79	0.128219	− 4189.83	− 4189.35	− 4189.74	0.112703
$F_{09}$	0	0	0	0	0	0	0	0
$F_{10}$	4.44E−16	4.44E−16	4.44E−16	2.96E−31	4.44E−16	4.44E−16	4.44E−16	2.96E−31
$F_{11}$	0	0	0	0	0	0	0	0
$F_{12}$	0.000216	0.001272	0.000658	0.000299	0.001703	1.168716	0.04321	0.20901
$F_{13}$	0.000511	0.007319	0.00297	0.001533	0.005932	0.039317	0.02102	0.009747
$F_{14}$	0.998004	2.982105	1.631052	0.603913	0.998005	3.26875	2.295492	0.764556
$F_{15}$	0.000396	0.002252	0.000918	0.000614	0.00034	0.000753	0.000556	0.000112
$F_{16}$	− 1.03163	− 1.03161	− 1.03162	4.97E−06	− 1.03163	− 1.03156	− 1.03161	1.77E−05
$F_{17}$	0.397902	0.39855	0.398055	0.000156	0.397898	0.398448	0.398754	0.000123
$F_{18}$	3.000002	3.002942	3.00114	0.000881	3.000092	3.012453	3.002965	0.002992
$F_{19}$	− 3.86262	− 3.67339	− 3.82681	0.062194	− 3.86133	− 3.35382	− 3.45615	0.00222
$F_{20}$	− 3.30347	− 2.99296	− 3.21544	0.087194	− 3.18619	− 2.09781	− 2.96355	0.22271
$F_{21}$	− 10.0703	− 4.94331	− 9.19656	1.177046	− 5.03931	− 4.65914	− 4.89076	0.108995
$F_{22}$	− 10.3638	− 4.97206	− 9.45097	1.24461	− 5.06156	− 4.68646	− 4.92932	0.081394
$F_{23}$	− 10.2523	− 9.1078	− 9.68354	0.321256	− 5.56499	− 3.7376	− 4.20326	0.252545

**Table 6:** Identified values of unknown parameters using MFHA and other state-of-art algorithms at 1073 K.

Algorithms	BES	AEO	DE	GWO	GWO-WOA	HBA	MRFO	PSO	WOA	FHA	SSA^21^	ISO^27^	CHDJ^20^	SOA^61^	ASO^21^	SCSO^23^	MFHA
$E_o (V)$	0.915	0.909	0.912	0.975	0.902	0.958	0.916	1.136	0.88	0.897	0.898	0.927	0.923	0.911	0.902	0.925	0.901
*A*(*V*)	8.71E−03	3.18E−04	5.12E−02	1.40E−03	8.23E−04	1.59E−03	1.39E−02	9.33E−02	4.67E−04	5.20E−02	1.78E−02	5.88E−02	1.23E−02	2.05E−03	2.94E−02	1.23E−02	9.23E−04
$I_{a.e}(mA/cm^2)$	70.53	12.82	97.53	70.01	75.06	98.33	92.49	21.38	98.56	74.76	89.6	29.9	100	48.1	47.7	100	25.29
$I_{c.e} (mA/cm^2)$	47.01	98	47.87	93.85	11.75	48.57	66.34	18.26	56.76	74.76	–	–	100	–	–	100	36.88
*B*(*V*)	0.33	0.98	0.89	0.25	0.93	0.71	0.01	0.77	0.53	0.74	0.07	0.6	4.45E−14	0	0.15	4.19E−14	0.59
$I_{lim}(mA/cm^2)$	321.02	1000	1529.46	6975.07	1626.53	6325.67	1857.49	5630.46	1114.93	7476.65	7390	5040	9283	6429	9389	9452	3402.21
$R_{ohm} (k\Omega .cm^2)$	9.28E−04	1.01E−03	3.37E−03	1.09E−04	4.42E−04	1.55E−04	1.02E−03	2.45E−04	1.10E−04	9.64E−04	8.77E−04	7.13E−04	1.02E−03	1.00E−04	7.30E−04	1.03E−03	9.23E−04
MSE	3.55E−06	3.31E−06	3.55E−06	3.20E−06	3.33E−05	3.93E−06	3.33E−06	6.10E−06	3.45E−06	4.07E−06	3.38E−06	3.42E−06	2.92E−06	4.12E−06	4.96E−06	2.92E−06	1.98E−06

**Table 7:** Identified values of unknown parameters using MFHA and other state-of-art algorithms at 1173 K.

Algorithms	BES	AEO	DE	GWO	GWO-WOA	HBA	MRFO	PSO	WOA	FHA	SSA^21^	ISO^27^	CHDJ^20^	SOA^61^	ASO^21^	SCSO^23^	MFHA
$E_o (V)$	0.913	0.899	0.891	0.941	0.953	0.941	0.899	0.729	0.899	0.879	0.881	0.8906	0.89	0.8795	0.881	0.89	0.931
*A*(*V*)	1.03E−04	3.96E−03	1.80E−04	1.04E−03	1.14E−04	1.03E−04	4.98E−04	2.71E−04	3.43E−04	2.11E−04	5.80E−04	1.76E−02	7.94E−17	7.77E−03	1.06E−02	2.51E−17	8.54E−04
$I_{a.e}(mA/cm^2)$	95.56	85.56	78.36	95.56	95.56	98.97	60.36	98.35	91.27	76.74	99	27.9	99.95	87.3	28.5	99.97	76.75
$I_{c.e} (mA/cm^2)$	76.56	41.98	72.48	48.78	78.24	57.98	58.56	76.56	54.76	57.98	–	–	94.3	–	–	83.15	27.75
*B*(*V*)	0.76	0.93	0.84	0.67	0.64	0.76	0.31	0.57	0.41	0.57	0.23	1.12	1	0.28	0.37	1	0.14
$I_{lim}(mA/cm^2)$	6789.94	5675.87	4555.45	6329.09	9981.73	9247.74	6684.07	9967.34	6789.94	4587.98	9499	2027	1939	9465	9422	1939	6989.56
$R_{ohm} (k\Omega .cm^2)$	4.45E−04	7.57E−04	6.63E−04	4.45E−04	2.03E−04	1.06E−04	6.56E−04	6.60E−04	6.45E−04	4.04E−04	2.90E−04	5.30E−04	6.93E−05	2.11E−04	5.34E−04	6.93E−05	8.45E−04
MSE	8.78E−06	8.23E−06	1.57E−05	9.23E−06	1.43E−05	1.40E−05	1.57E−05	1.29E−04	8.23E−06	2.98E−06	2.10E−06	3.17E−06	1.46E−06	3.16E−06	6.71E−06	1.46E−06	1.28E−06

**Table 8:** Identified values of unknown parameters using MFHA and other state-of-art algorithms at 1213 K.

Algorithms	BES	AEO	DE	GWO	GWO-WOA	HBA	MRFO	PSO	WOA	FHA	SSA^21^	ISO^27^	CHDJ^20^	SOA^61^	ASO^21^	SCSO^23^	MFHA
$E_o (V)$	0.982	0.873	0.912	0.943	0.981	0.912	0.877	1.2	0.912	0.795	0.857	0.862	0.862	0.856	0.837	0.862	0.791
*A*(*V*)	1.40E−01	8.70E−02	8.75E−02	6.94E−02	8.24E−02	8.75E−02	1.67E−04	6.54E−02	8.75E−02	3.07E−04	2.01E−03	1.00E−02	4.26E−17	3.12E−02	4.70E−03	2.69E−18	1.03E−04
$I_{a.e} (mA/cm^2)$	79.89	78.85	67.58	56.022	49.97	76.56	99.99	32.22	93.48	66.69	91	30	61.82	47.8	61	97.75	76.13
$I_{c.e} (mA/cm^2)$	68.87	47.63	46.57	44.14	40.41	45.87	87.98	8.36	47.76	65.87	–	–	20.93	–	–	36.36	74.36
*B*(*V*)	0.81	0.63	0.75	0.13	0.26	0.67	0.77	0.15	0.65	0.47	0.3	0.42	0.57	0.41	0.06	0.57	0.83
$I_{lim}(mA/cm^2)$	1422.25	4563.67	6787.87	3678.07	2351.32	8751.27	5970.52	9786.87	9967.73	5798.83	9633	1091	1307	1000	5588	1307.5	5599.92
$R_{ohm} (k\Omega .cm^2)$	2.66E−04	6.58E−04	5.04E−04	4.05E−04	2.05E−04	3.07E−04	6.91E−04	1.04E−04	1.93E−04	1.65E−04	1.07E−04	2.31E−04	3.93E−18	1.00E−04	1.21E−04	4.23E−18	1.63E−04
MSE	1.47E−05	1.42E−05	1.44E−05	1.19E−05	1.56E−05	1.40E−05	1.42E−05	1.29E−04	1.19E−05	2.05E−06	3.02E−06	5.02E−06	1.90E−06	4.38E−06	2.87E−05	1.90E−06	1.41E−06

**Table 9:** Identified values of unknown parameters using MFHA and other state-of-art algorithms at 1273 K.

Algorithms	BES	AEO	DE	GWO	GWO-WOA	HBA	MRFO	PSO	WOA	FHA	SSA^21^	ISO^27^	CHDJ^20^	SOA^61^	ASO^21^	SCSO^23^	MFHA
$E_o (V)$	0.864	0.921	0.877	0.878	0.901	0.913	0.884	0.921	0.877	0.915	0.845	0.842	0.848	0.856	0.848	0.848	0.913
*A*(*V*)	4.86E−02	7.69E−02	7.90E−02	8.34E−02	8.17E−02	5.75E−02	9.50E−02	6.63E−02	7.91E−02	4.06E−04	1.30E−03	1.00E−02	6.45E−17	3.12E−02	1.50E−03	4.98E−17	2.63E−04
$I_{a.e}(mA/cm^2)$	98.9	87.52	95.67	97.94	94.37	67.93	91.73	75.89	98.65	76.39	42.3	30	99.1	47.8	37.7	96.15	47.57
$I_{c.e}(mA/cm^2)$	87.68	63.85	63.84	93.45	56.73	41.36	47.85	27.94	37.87	37.93	–	–	66.85	–	–	28.17	21.56
*B*(*V*)	0.97	0.38	0.57	0.29	0.67	0.59	0.57	0.59	0.83	0.54	0.17	0.29	0.63	0.4	0.13	0.63	0.65
$I_{lim}(mA/cm^2)$	9985.84	6793.84	6364.16	9662.25	9479.97	8739.69	8794.82	1663.67	6784.69	4978.84	8772	9690	1530	1000	7638	1530.8	5228.86
$R_{ohm} (k\Omega .cm^2)$	2.79E−04	4.06E−04	3.34E−04	5.57E−04	4.56E−04	5.05E−04	6.07E−04	3.87E−04	1.65E−04	3.58E−04	4.88E−04	4.88E−04	1.90E−19	1.00E−04	2.20E+04	2.11E−19	2.63E−04
MSE	9.80E−06	1.19E−05	9.78E−06	1.04E−05	1.19E−05	9.78E−06	1.08E−05	9.53E−05	1.42E−05	2.44E−06	2.71E−06	5.87E−06	2.46E−06	4.38E−06	7.46E−06	2.46E−06	1.91E−06

**Table 10:** Statistical analysis and non-parametric test at 1073 K.

Statistical analysis based on objective function (SSE)											
Algorithm	BES	AEO	DE	GWO	GWO-WOA	HBA	MRFO	PSO	WOA	FHA	MFHA
SSE	7.09E−05	6.61E−05	7.09E−05	6.39E−05	6.66E−04	7.86E−05	6.66E−05	0.000122	6.89E−05	8.13E−05	1.04E−05
Max	9.87E−04	4.66E−04	8.77E−04	9.87E−04	8.10E−04	7.09E−04	4.09E−04	1.94E−03	9.89E−04	3.09E−04	3.30E−05
Min	7.09E−05	6.61E−05	7.09E−05	6.39E−05	6.66E−04	7.86E−05	6.66E−05	0.000122	6.89E−05	8.13E−05	1.04E−05
Mean	2.84E−04	1.69E−04	4.35E−04	5.57E−04	3.14E−04	1.81E−04	1.22E−04	7.16E−04	2.54E−04	1.09E−04	1.79E−05
Standard Deviation	2.43E−04	1.18E−04	2.51E−04	3.25E−04	2.58E−04	1.69E−04	7.75E−05	6.95E−04	2.37E−04	4.77E−05	4.26E−06
FriedMan’s Rank and Wilcoxon Test											
Friedman’s Rank	6.645165	5.946882	8.89084	9.498222	6.890946	6.403926	5.657167	9.252042	6.833614	5.241886	1.421761
Rank	6	4	9	11	8	5	3	10	7	2	1
R+	465	465	465	465	465	465	465	465	465	465	
R−	0	0	0	0	0	0	0	0	0	0	
p-Value	3.00E−11	2.99E−11	2.94E−11	2.99E−11	2.97E−11	2.95E−11	2.97E−11	2.91E−11	2.99E−11	2.93E−11	
Result	+	+	+	+	+	+	+	+	+	+

**Table 11:** Statistical analysis and non-parametric test at 1173 K.

Statistical analysis based on objective function (SSE)											
Algorithm	BES	AEO	DE	GWO	GWO-WOA	HBA	MRFO	PSO	WOA	FHA	MFHA
SSE	3.51E−04	3.29E−04	6.28E−04	3.69E−04	5.71E−04	5.61E−04	6.29E−04	5.17E−03	3.29E−04	1.99E−05	2.30E−05
Max	8.71E−03	9.53E−03	9.52E−03	9.79E−03	9.85E−03	9.52E−03	9.83E−03	9.78E−02	9.61E−03	9.48E−04	9.62E−05
Min	3.51E−04	3.29E−04	6.28E−04	3.69E−04	5.71E−04	5.61E−04	6.29E−04	5.17E−03	3.29E−04	1.99E−05	2.30E−05
Mean	3.63E−03	4.81E−03	4.71E−03	4.55E−03	4.61E−03	5.45E−03	5.40E−03	1.71E−02	3.12E−03	2.12E−04	5.97E−05
Standard deviation	2.51E−03	3.16E−03	3.20E−03	3.39E−03	3.69E−03	3.44E−03	3.70E−03	2.47E−02	3.28E−03	2.45E−04	8.70E−05
FriedMan’s Rank and Wilcoxon Test											
Friedman’s Rank	6.548057	7.605792	6.993653	7.913376	7.865693	7.630874	7.878498	9.513548	6.124174	3.098222	1.22428
Rank	4	6	5	10	8	7	9	11	3	2	1
R+	465	465	465	465	465	465	465	465	465	465	
R−	0	0	0	0	0	0	0	0	0	0	
p-Value	2.84E−11	2.86E−11	2.85E−11	2.86E−11	2.85E−11	2.83E−11	2.84E−11	2.85E−11	2.86E−11	7.09E−08	
Result	+	+	+	+	+	+	+	+	+	+

**Table 12:** Statistical analysis and non-parametric test at 1213 K.

Statistical analysis based on objective function (SSE)											
Algorithm	BES	AEO	DE	GWO	GWO-WOA	HBA	MRFO	PSO	WOA	FHA	MFHA
SSE	5.87E−04	5.66E−04	5.77E−04	4.77E−04	6.25E−04	5.60E−04	5.66E−04	5.17E−03	4.77E−04	8.19E−05	1.03E−05
Max	9.39E−03	9.85E−03	9.88E−03	6.86E−03	6.93E−03	8.00E−03	9.58E−03	3.00E−02	9.95E−03	9.97E−04	6.39E−05
Min	5.87E−04	5.66E−04	5.77E−04	4.77E−04	6.25E−04	5.60E−04	5.66E−04	5.17E−03	4.77E−04	8.19E−05	1.03E−05
Mean	3.71E−03	5.34E−03	4.96E−03	1.98E−03	3.61E−03	3.99E−03	3.30E−03	1.58E−02	4.56E−03	7.06E−04	3.87E−05
Standard deviation	3.13E−03	3.43E−03	3.39E−03	2.20E−03	2.36E−03	3.04E−03	3.20E−03	9.57E−03	3.72E−03	3.72E−04	3.76E−06
FriedMan’s Rank and Wilcoxon Test											
Friedman’s Rank	6.414724	8.439125	7.293653	5.580043	6.932359	6.630874	6.445165	10.31355	8.024174	5.164889	1.157613
Rank	4	10	8	3	7	6	5	11	9	2	1
R+	465	465	465	465	465	465	465	465	465	465	
R−	0	0	0	0	0	0	0	0	0	0	
p-Value	2.89E−11	2.93E−11	2.97E−11	2.96E−11	2.97E−11	2.96E−11	2.96E−11	2.97E−11	2.96E−11	2.95E−11	
Result	+	+	+	+	+	+	+	+	+	+

**Table 13:** Statistical analysis and non-parametric test at 1273 K.

Statistical analysis based on objective function (SSE)											
Algorithm	BES	AEO	DE	GWO	GWO-WOA	HBA	MRFO	PSO	WOA	FHA	MFHA
SSE	3.92E−04	4.77E−04	3.91E−04	4.14E−04	4.77E−04	3.91E−04	4.33E−04	3.81E−03	5.69E−04	9.76E−05	1.60E−05
Max	9.84E−03	6.45E−02	6.38E−03	6.94E−03	8.63E−03	7.21E−03	7.16E−03	1.87E−02	9.59E−03	2.36E−03	2.87E−05
Min	3.92E−04	4.77E−04	3.91E−04	4.14E−04	4.77E−04	3.91E−04	4.33E−04	3.81E−03	5.69E−04	9.76E−05	1.60E−05
Mean	3.89E−03	5.76E−03	1.82E−03	2.53E−03	2.93E−03	3.05E−03	2.08E−03	7.22E−03	2.69E−03	5.39E−04	2.02E−05
Standard deviation	2.56E−03	1.12E−02	2.02E−03	2.53E−03	2.76E−03	2.59E−03	2.40E−03	4.94E−03	3.12E−03	5.97E−04	5.09E−06
FriedMan’s Rank and Wilcoxon Test											
Friedman’s Rank	8.348057	8.105792	5.36032	7.780043	7.465693	6.69754	5.945165	9.380215	7.59084	4.564889	1.157613
Rank	10	9	3	8	6	5	4	11	7	2	1
R+	465	465	465	465	465	465	465	465	465	465	
R−	0	0	0	0	0	0	0	0	0	0	
p-Value	1.95E−11	1.95E−11	1.94E−11	1.95E−11	1.95E−11	1.95E−11	1.94E−11	1.95E−11	1.94E−11	1.95E−11	
Result	+	+	+	+	+	+	+	+	+	+

**Table 14:** Identified values of unknown parameters and objective function using MFHA at different temperatures.

Temperature (K)	$E_o (V)$	*A*(*V*)	$I_{a.e}(mA/cm^2)$	$I_{c.e} (mA/cm^2)$	*B*(*V*)	$I_{lim}(mA/cm^2)$	$R_{ohm} (k\Omega \;cm^2)$	SSE
873 K	1.122	5.64E−02	12.47	5.96	0.148	2052	7.31E−03	1.18E−03
923 K	1.120	5.94E−02	17.52	6.26	0.052	1569	6.08E−03	6.12E−03
973 K	1.121	5.49E−02	23.56	75.66	0.051	1567	5.18E−03	2.21E−02
1023 K	1.116	4.63E−02	27.52	7.81	0.061	1595	4.46E−03	5.18E−02
1073 K	1.115	3.61E−02	27.53	6.67	0.065	1599	3.97E−03	6.00E−02

**Table 15:** Identified values of unknown parameters and objective function using MFHA at different pressures.

Pressure (atm)	$E_o (V)$	*A*(*V*)	$I_{a.e}(mA/cm^2)$	$I_{c.e} (mA/cm^2)$	*B*(*V*)	$I_{lim}(mA/cm^2)$	$R_{ohm} (k\Omega \; cm^2)$	SSE
1 atm	1.078	3.61E−02	27.52	6.66	0.065	1599	3.97E−03	6.05E−02
2 atm	1.104	3.58E−02	27.41	6.65	0.066	1598	3.96E−03	6.11E−02
3 atm	1.115	3.62E−02	27.52	6.65	0.065	1599	3.96E−03	5.53E−02
4 atm	1.122	3.61E−02	27.71	6.58	0.064	1593	3.97E−03	5.11E−02
5 atm	1.127	3.61E−02	27.53	6.67	0.065	1599	3.96E−03	6.64E−02

## Results and discussions

**Fig. 5:**
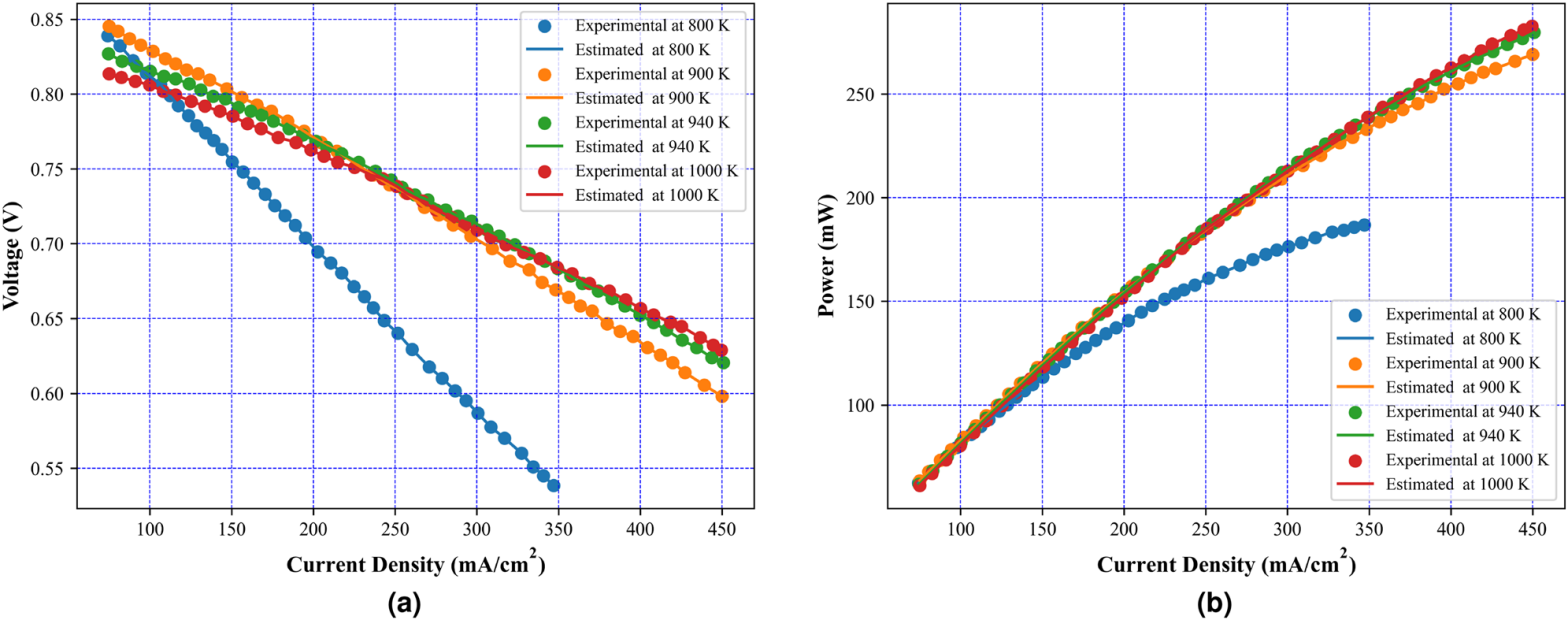
Experimental and estimated polarization characteristics using MFHA (**a**) I–V characteristics, (**b**) I–P characteristics.

**Fig. 6:**
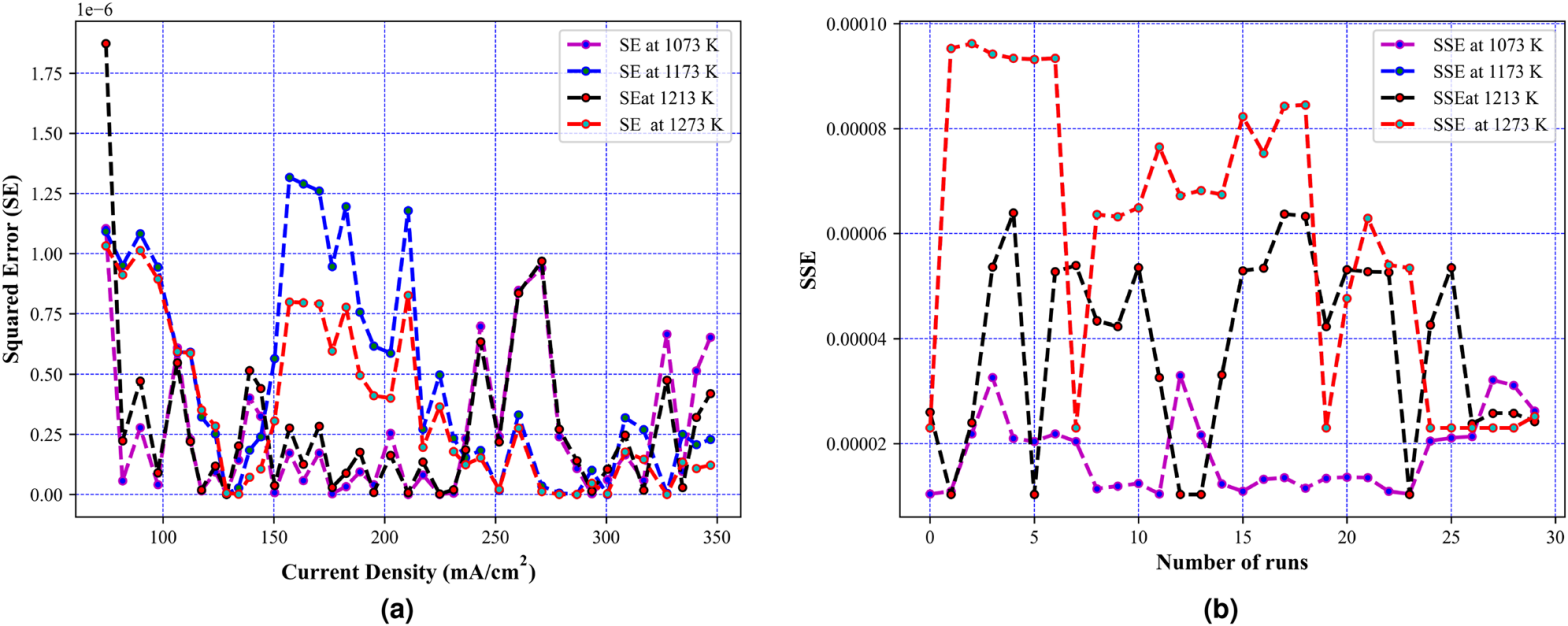
Errors obtained at different temperatures using MFHA (**a**) variation of squared error with current density, and (**b**) variation of sum of squared error with number of runs.

**Table 16:** Analysis of MFHA with different scenarios such as number of data points, temperatures and pressures.

Number of data points	SSE value	Standard deviation	Temperatures	SSE value	Pressure	SSE value
38	1.03E−05	3.76E−06	873	1.18E−03	1 atm	6.05E−02
39	1.04E−05	4.26E−06	923	6.12E−03	2 atm	6.11E−02
41	1.60E−05	5.09E−06	973	2.21E−02	3 atm	5.53E−02
43	2.30E−05	8.70E−06	1023	5.18E−02	4 atm	5.11E−02
			1073	6.00E−02	5 atm	6.64E−02

**Fig. 7:**
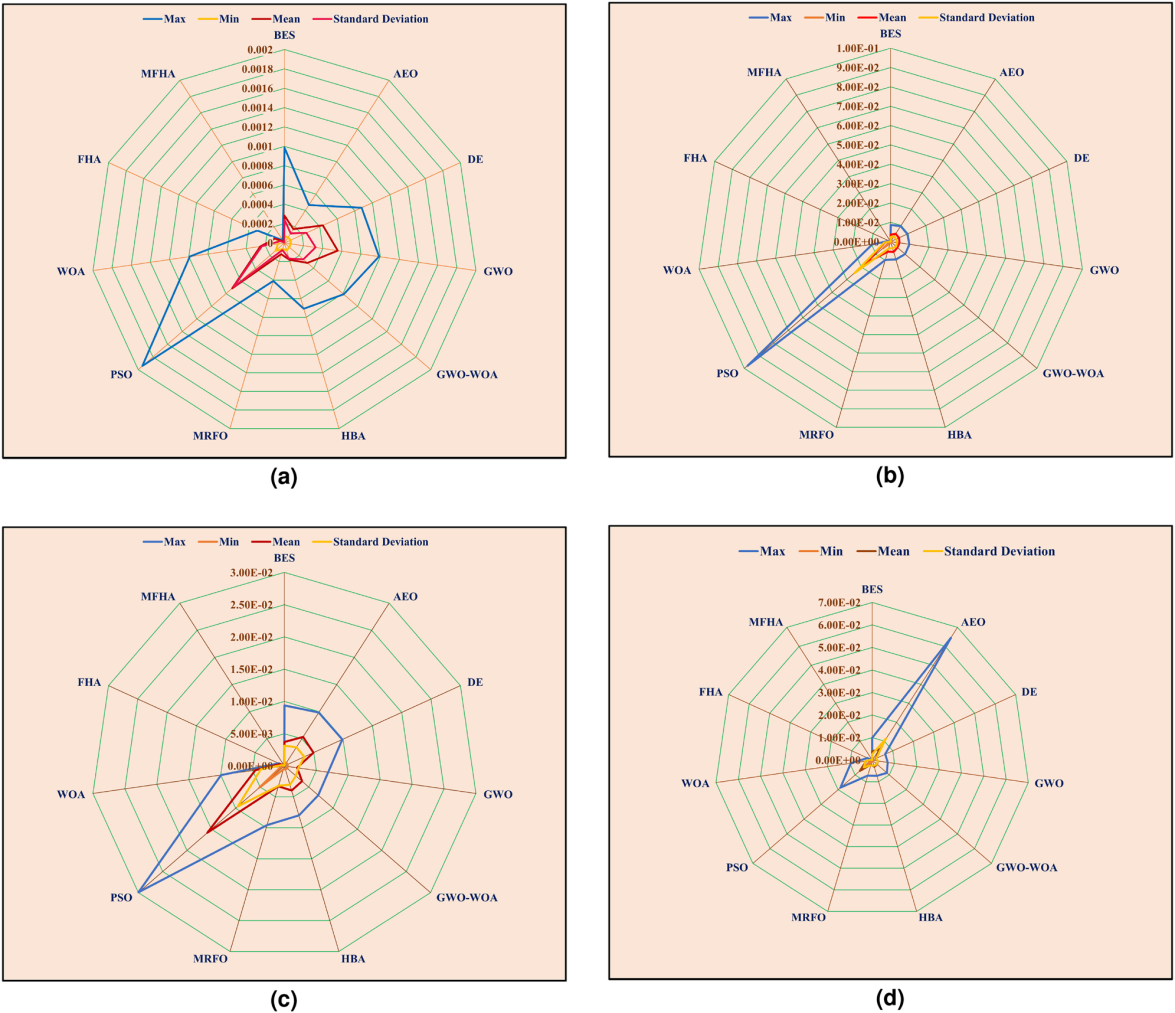
Comparison of statistical indices on radar chart at (**a**) 1073 K, (**b**) 1173 K, (**c**) 1213 K, and (**d**) 1273 K.

**Fig. 8:**
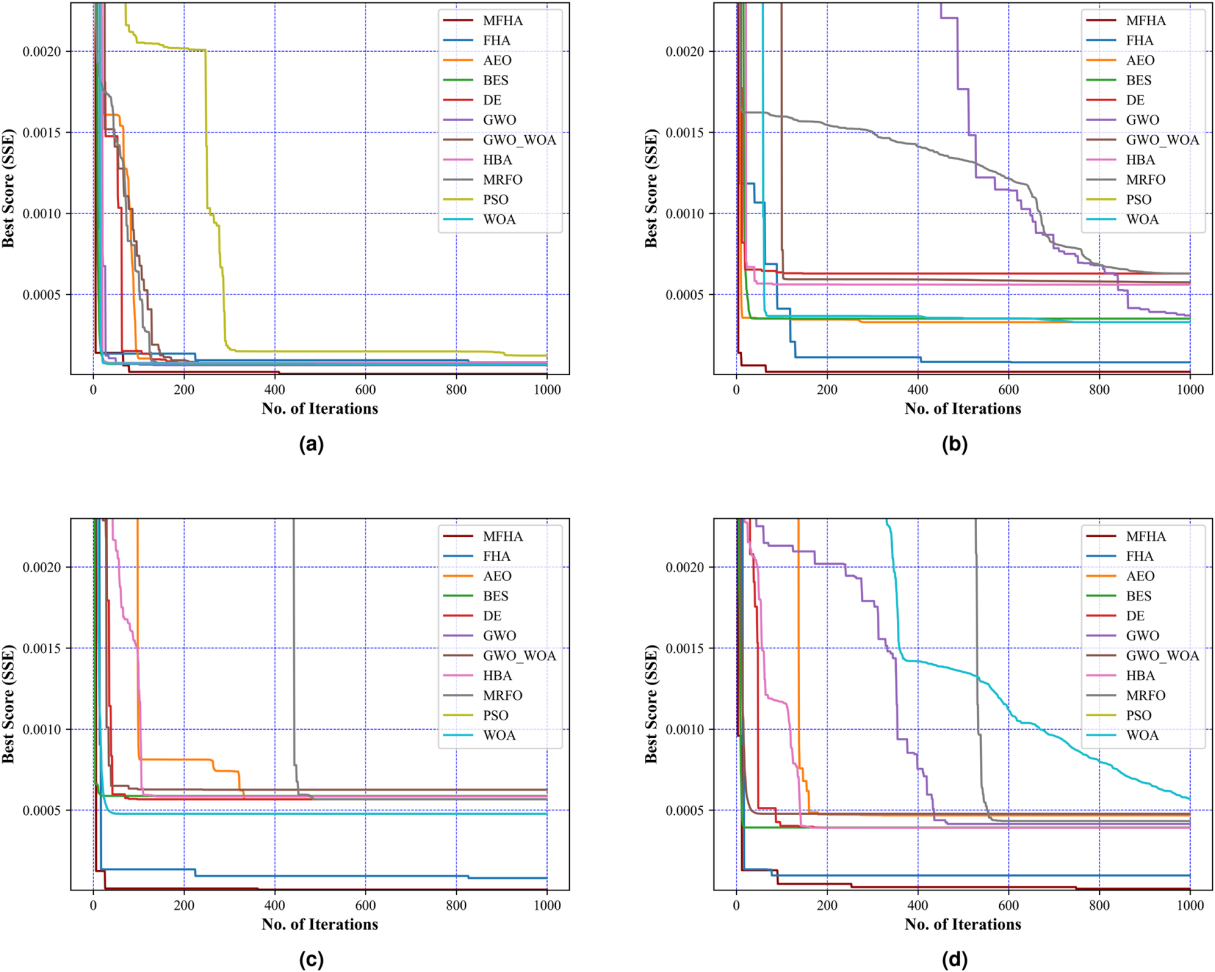
Convergence curves at (**a**) 1073 K, (**b**) 1173 K, (**c**) 1213 K, and (**d**) 1273 K.

**Fig. 9:**
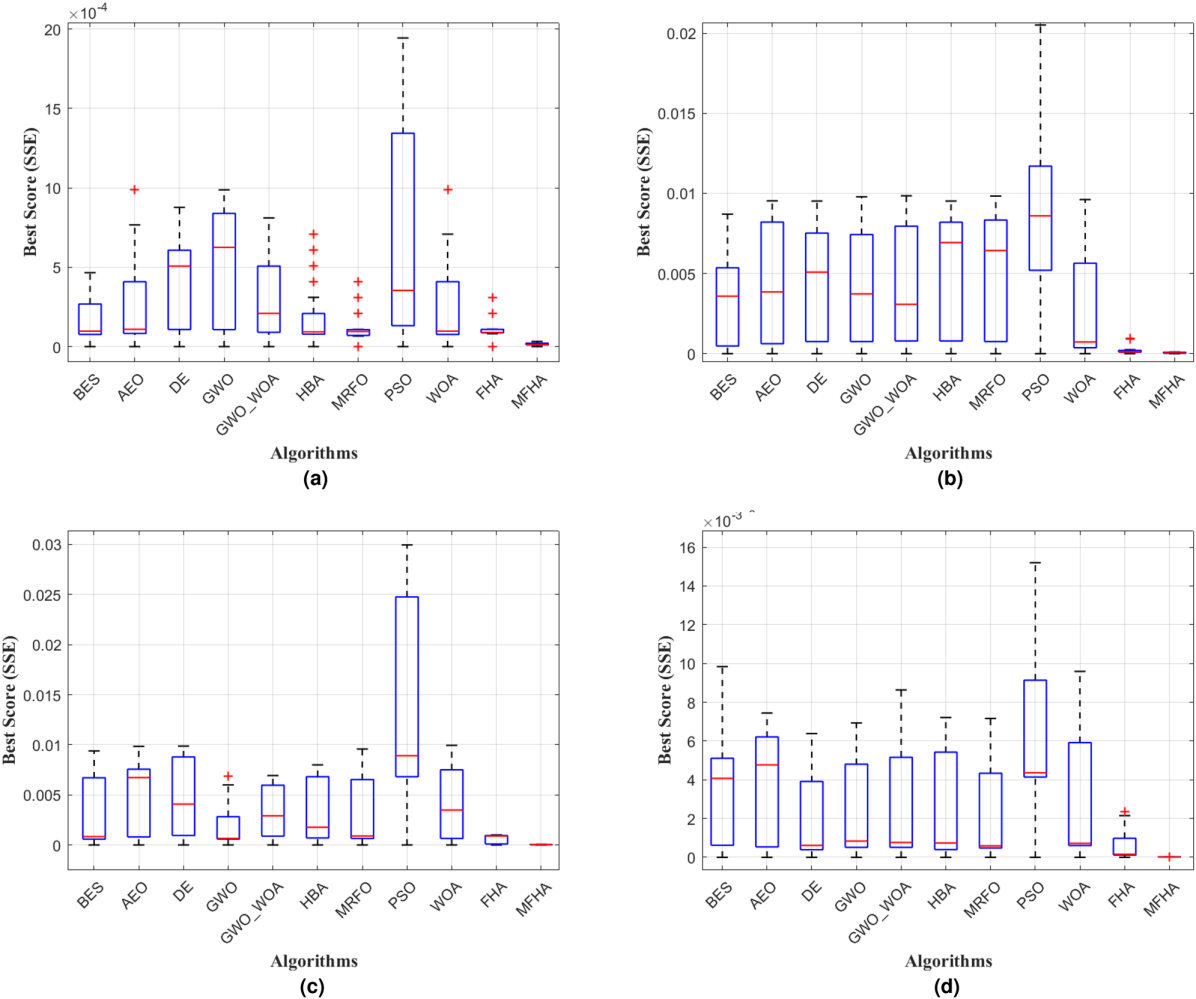
Boxplot at (**a**) 1073 K, (**b**) 1173 K, (**c**) 1213 K, and (**d**) 1273 K.

**Fig. 10:**
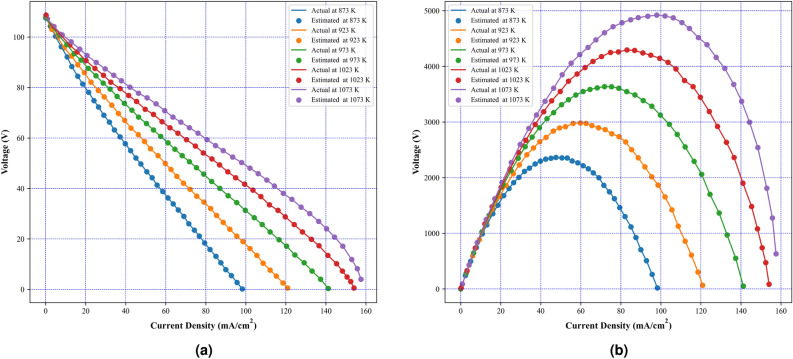
Experimental and estimated polarization characteristics using MFHA at different temperatures (**a**) I–V characteristics, (**b**) I–P characteristics.

In this paper, a modified version of the Fire Hawk technique is put forth to estimate the values of unknown parameters of SOFC. The modified version of fire hawk algorithm provides better convergence and avoids the local optima trap by using two factor of improvements $Ir_1$ and $Ir_2$. $Ir_1$ linearly decreases from 1 to 0 which varies over iterations and controls the position of global best position whereas $Ir_2$ regulates the position of fire hawk. These factors are applied in updating the position of fire hawks and preys inside and outside of the territory of each fire hawk. The flowchart for the working of MFHA is presented as Fig. 4. Firstly, the developed MFHA is validated on twenty-three benchmark functions and its performance is compared with original FHA. After, evaluating MFHA’s effectiveness, it is implemented on problem of SOFC model parameter identification. To prove the authenticity of MFHA, two different case studies have been considered consisting of two different datasets of SOFC polarization characteristics. Case study I consists of a commercially available real power enhanced cylindrical cell developed by Siemens^21^ and case study II considers a theoretical 5 kW, 96 cells SOFC stack^62^. Case study 1 consists of a data set of polarization curves at four different temperatures viz 1073 K, 1173 K, 1213 K, and 1273 K whereas, case study II considers two different data sets of polarization curves at different pressures and temperatures. Both case studies use upper and lower boundaries of unknown parameters as tabulated in Table 5 and have been taken from^21^. An objective function is considered to obtain the accurate polarization characteristics and is expressed as in Eq. (7) and is subjected to variable constraints as mentioned in Eqs. (8)–(10). The decision variables $E_{o}$, *A*, $I_{a.e}$, $I_{c.e}$, *B*, $I_{lim}$, and $R_{ohm}$ need to be determined optimally to obtain the minimum value of objective function. The primary goal of this study is to minimize the error between the experimental and estimated polarization characteristic and obtain an optimal value of unknown parameters to produce an accurate SOFC mathematical model. Therefore, a new MFHA is developed to identify unknown parameters. The proposed parameter identification algorithm is implemented using MATLAB 2022b on a 5.2 GHz Intel core i7 computer with 8.0 GB RAM. The performance of developed MFHA is compared with original FHA as well as well-established algorithms and algorithms from literature that are applied in SOFC parameter identification problem. In literature, the mean squared error (MSE) is used as a performance criteria. Therefore, for a fair comparison, MSE is also presented to prove its superiority. The simulation settings of well-established algorithms are set as 30 independent runs and 1000 iterations with 30 search agents. The best value among 30 runs is recorded as obtained value of objective function i.e. SSE. At this value of the objective function, the corresponding values of parameters are regarded as optimum parameters. The unknown parameters obtained with respect to this objective function value are considered as optimal parameters. The accuracy of the algorithm is identified by mapping the polarization characteristics. A good identifier is one with great closeness between experimental and estimated polarization characteristics. Therefore, polarization characteristics considering, I–V and I–P curves are presented in this study to show the accuracy of MFHA in identifying the unknown parameters. MFHA and other algorithms used in this study are metaheuristic algorithms and exhibit random behavior in finding an optimal solution. Therefore, statistical analysis is performed to evaluate the robustness of the developed algorithm in terms of mean, maximum (max), minimum (min), and standard deviation of objective function value i.e. SSE over 30 runs. Box plot analysis, considering interquartile range and median, is also presented to validate the robustness of MFHA over other algorithms. Additionally, a comparison of statistical indices is also presented on radar chart to illustrate their variation for different algorithms. Moreover, the speed of an algorithm in reaching to optimal solution, without trapping in local minima is another criteria of evaluating its performance. Thus, convergence analysis is also presented and compared with other algorithms. The results considering benchmark evaluations and performance of MFHA in identifying unknown parameters for both case studies are given in next subsections.

**Fig. 11:**
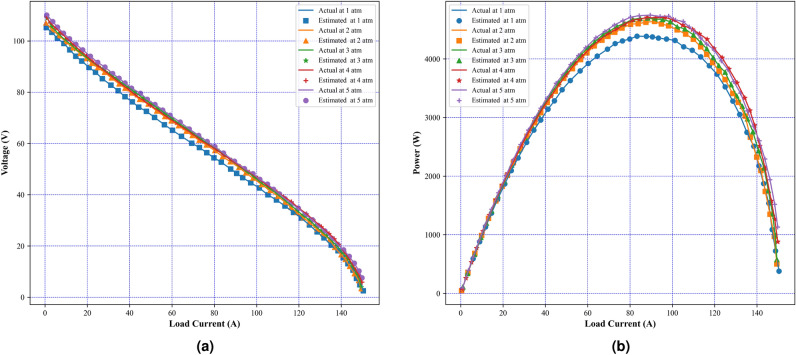
Experimental and estimated polarization characteristics using MFHA at different pressure (**a**) I–V characteristics, (**b**) I–P characteristics.

**Fig. 12:**
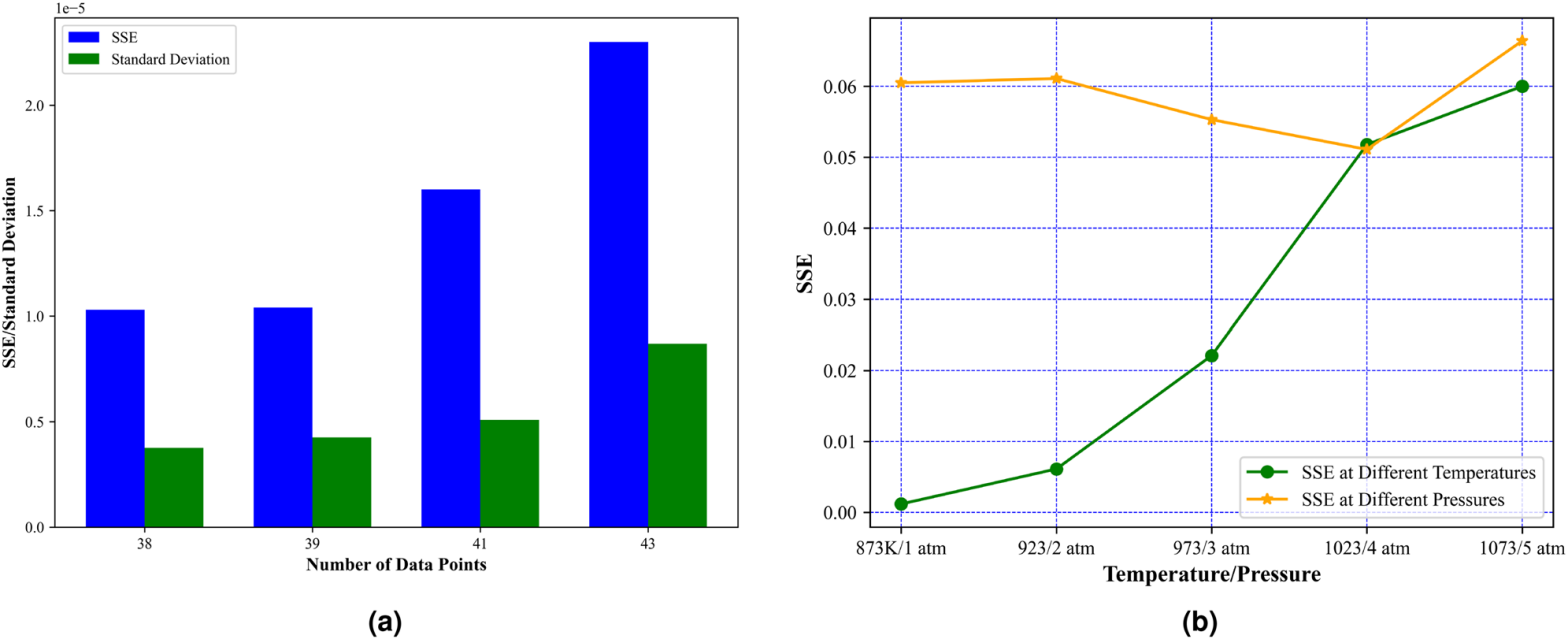
Variation of objective function (SSE) for MFHA with (**a**) number of data points, and (**b**) different scenarios of temperatures and pressures.

**Table 17:** Comparison of MFHA with other state-of-the-art algorithms from literature.

Algorithms	Temperature					Pressure				
	873 K	923 K	973 K	1023 K	1073 K	1 atm	2 atm	3 aatm	4 atm	5 atm
MSMPA^21^	3.28E−03	4.95E−03	7.26E−03	9.65E−03	3.65E−02	6.16E−02	7.68E−02	8.65E−02	1.29E−01	3.54E−01
CBSO^25^	5.50E−02	6.01E−02	8.71E−02	1.07E−01	5.41E−01	1.76E+00	2.23E+00	2.37E+00	2.51E+00	2.64E+00
IRFO^63^	4.18E−03	5.44E−03	7.65E−03	5.16E−02	6.38E−02	8.14E−02	9.65E−02	2.66E−01	4.46E−01	5.65E−01
SBO^64^	3.21E−02	4.26E−02	6.24 E−02	7.62E−02	1.84E−01	1.11E+00	1.42E+00	1.62E+00	2.27E+00	2.62E+00
COA^30^	7.23E−03	8.32E−03	1.54E−02	4.03E−02	6.24E−02	3.06E−01	4.34E−01	6.03E−01	8.25E−01	8.66E−01
CGWO^16^	8.20E−04	7.66E−04	6.92E−03	8.64E−03	2.56E−02	6.24E−02	8.62E−02	1.38E−01	4.25E−01	5.72E−01
FHA	2.97E−03	6.78E−03	2.87E−02	6.12E−02	6.64E−02	6.21E−02	6.78E−02	6.97E−02	5.84E−02	7.01E−02
MFHA	1.18E−03	6.12E−03	2.21E−02	5.18E−02	6.00E−02	6.05E−02	6.11E−02	5.53E−02	5.11E−02	6.64E−02

### Benchmark evaluations with modified fire Hawk algorithm

To prove the superiority of MFHA over FHA, its effectiveness is evaluated on twenty-three classical benchmark functions. The performance of MFHA is assessed for independent 30 runs and 1000 iterations. The information about functions, ranges, dimensions, and minimum values are tabulated in Table 3. The effectiveness of MFHA is evaluated based on maximum value (max), minimum value (min), standard deviation, and mean. A fair comparison between MFHA and FHA based on statistical evaluation of these functions is presented in Table 5. It is concluded from Table 5 that on the basis of min, max, mean and standard deviation, MFHA outperforms the original FHA. Also, MFHA performed better for $F_{01}$, $F_{02}$, $F_{03}$, $F_{04}$, $F_{05}$, $F_{07}$, $F_{08}$, $F_{15}$, $F_{17}$, $F_{19}$, $F_{21}$, $F_{22}$, and $F_{23}$ whereas for $F_{09}$, $F_{10}$, and $F_{11}$, the results are same as obtained using original FHA. For $F_{06}$, $F_{12}$, $F_{13}$, $F_{14}$, $F_{16}$, $F_{18}$, and $F_{20}$, MFHA performed worst. This analysis concludes that MFHA outperformed original FHA for most of the benchmark functions. Therefore, MFHA is found to be robust algorithm and can be applied to the problem of parameter identification of SOFC.

### Case study I

After the validation of results on benchmark evaluations, the MFHA is used for the identification of parameters of SOFC. In this study, a commercially available SOFC developed by Siemens^21^ has been taken into consideration to extract the experimental data sets at four different temperatures. The boundary limits for the unknown parameters are mentioned in Table 2. Results obtained using MFHA have been compared with those of FHA and other well-established algorithms such as honey badger algorithm (HBA)^59^, particle swarm optimization (PSO)^53^, artificial ecosystem optimization (AEO)^58^, grey wolf optimization (GWO)^55^, whale optimization algorithm^56^, differential evolution (DE)^54^, bald eagle search (BES)^52^, manta ray foraging optimization (MRFO)^57^, and hybrid grey wolf-whale optimization algorithm (GWO-WOA)^60^. The algorithms that are considered from literature are salp swarm algorithm^21^, internal search optimization (ISO)^27^, seagull optimization algorithm (SOA)^61^, atomic search optimization (ASO)^21^, competitive hybrid differential evolution and jaya algorithm (CHDJ)^20^, and simplified competitive swarm optimizer (SCSO)^23^. The different parameters setting for these algorithms are presented in Table 4. The accuracy of the developed algorithm is identified by mapping polarization characteristics. These polarization characteristics are obtained at four different temperatures viz. 1073 K, 1173 K, 1213 K, and 1273 K. From Fig. 5, it is observed that polarization curves obtained from the experimental data points at all four temperatures perfectly match with the estimated data points using MFHA. Therefore, a good closeness of estimated I-V, and P-V curves with experimental ones at all four temperatures confirms the accuracy of MFHA in identification of unknown parameters of SOFC and proves that MFHA is a good identifier. The obtained value of unknown parameters and MSE are presented in Tables 6, 7, 8, and 9 for 1073 K, 1173 K, 1213 K, and 1273 K respectively. From results tabulated in these tables, it is observed that the lowest values of MSE over 30 runs for MFHA at 1073 K, 1173 K, 1213 K, and 1273 K are 1.98E−06, 1.28E−06, 1.41E−06, and 1.91E−06 respectively. It can also be concluded from these observations that MFHA outperforms among other algorithms and obtains optimal value of unknown parameters. To obtain and provide unbiased results and a fair comparison, statistical analysis considering the objective function i.e. SSE is also performed. Statistical indices such as mean, minimum, standard deviation, and maximum value of objective function (SSE) are used as specific metrics to demonstrate the performance of MFHA in comparison to other algorithms. In this work, the SSE is used as the objective function and the performance of MFHA is evaluated and compared with other algorithms based on 30 independent runs. Key statistical indices, including the minimum, maximum, mean, and standard deviation of SSE values, are calculated from these runs. The minimum SSE value represents the best performance, while the maximum SSE value represents the worst case performance. The mean provides a measure of central value and denotes the overall accuracy. From comparison view of point, it can be easily assessed lower and higher central points of used algorithms. Lower is central point of SSE values, more superior is the algorithm. The standard deviation shows the spread of 30 SSE values around the mean and shows variability. Lower is the standard deviation, more efficient is the algorithm. From the results mentioned in Tables 10, 11, 12, and 13, it is observed that MFHA outperformed other algorithms with the least value of SSE for all four different temperatures. The values of SSE at 1073 K, 1173 K, 1213 K, and 1273 K are found to be 1.04E−05, 2.30E−05, 1.03E−05, and 1.60E−05 respectively. Moreover, a pair-wise comparison is also performed between MFHA and other algorithms that are used for the identification of SOFC parameters. This comparison is based on the non-parametric Wilcoxon signed rank test, $R+$, $R-$, and $p-$ values. From Tables 10, 11, 12, and 13, it can be observed that MFHA obtained first rank and outperformed among all other algorithms at different temperatures. It wins over all other algorithms in all cases using Wilcoxon test which validates its reliability in solving the problem of PEMFC parameter extraction. Furthermore, from Fig. 6a, it is observed that there is very little variation of squared error (SE) at different current densities. Also, from Fig. 6b, it is observed that there is very little variation in SSE among 30 independent runs of MFHA which further confirms its robustness and reliability. Moreover, from Fig. 7, a comparison of different statistical indices observed in radar chart concludes that MFHA outperformed among other algorithms and shows its robustness in identifying parameters at different temperatures. The convergence curve and box plot analysis are also demonstrated to show the speed of convergence and robustness of MFHA. From Fig. 8, it is observed that MFHA converges faster and avoids local minima traps for all four different temperatures whereas, other algorithms stuck in the local minima. Therefore, MFHA shows faster convergence in comparison to other algorithms. Fig. 9, presents the box plot study at all different temperatures and it is observed that MFHA outperforms all other algorithms with small interquartile range and least median which further confirms the robustness and reliability of MFHA.

### Case study II

Case study II considers physics based dynamic tubular stack having 96 cells and 5 kW rating. In this case study, the effectiveness of MFHA is evaluated at two different scenarios of different temperatures and pressures. Firstly, the performance of MFHA is illustrated at five different temperatures viz 873 K, 923 K, 973 K, 1023 K and 1073 K whereas, the pressure of the reactants is considered as 3 atm. Secondly, the performance of MFHA is further evaluated at different pressures viz 1 atm, 2 atm, 3 atm, 4 atm, and 5 atm whereas, temperature is considered as 1073 K. The boundary limits for unknown parameters are considered from Table 2. The actual polarization characteristics have been taken from^23,62^. A comparison with state-of-art algorithms such as multi-swarm marine predator (MSMPA)^21^, chaotic binary shark smell optimizer (CBSO)^25^, improved red fox optimization (IRFO)^63^, Satin bowerbird optimizer (SBO)^64^, cyote optimization algorithm (COA)^30^, chaotic grey wolf optimization (CGWO)^16^, and original FHA is also presented in Table 17. From Fig. 10a,b, it can be noticed that both estimated I–V and I–P curves are in good coincidence with actual ones at different temperatures. Therefore, it is concluded from Fig. 10, that MFHA accurately identifies the unknown parameters of SOFC at different temperatures. and it is observed that MFHA outperforms among state-of-the-art algorithms and original FHA in almost all scenarios except at 873 K and 923 K from CGWO algorithm.Further, Table 14, tabulates the obtained values of unknown parameters and SSE at different temperatures. The obtained values of SSE at 873 K, 923 K, 973 K, 1023 K, and 1073 K are 1.18E−03, 6.12E−03, 2.21E−02, 5.18E−02, and 6.00E−02 respectively. The performance of MFHA at different pressures is also evaluated and the results are tabulated in Table 15. The obtained values of SSE at 1 atm, 2 atm, 3 atm, 4 atm, and 5 atm are 6.05E−02, 6.11E−02, 5.53E−02, 5.11E−02, and 6.64E−02 respectively. Moreover, from comparison presented in Table 17, it is observed that MFHA outperforms among state-of-the-art algorithms and original FHA in almost all scenarios except at 873 K and 923 K from CGWO algorithm. From Fig. 11a,b, it is observed that estimated I–V and I–P curves accurately matches with actual ones and prove the effectiveness of MFHA in determining the unknown parameter values even at different pressures.

## Sensitivity analysis

In this section the performance of MFHA is analysed with different number of scenarios such as number of data points, non-linearity of data at different temperatures and pressures. This analysis is divided into two scenarios viz based on number of data points for case study I and non-linearity of data points for case stud II. Case study I consists of four different experimental I–V data points for a single solid oxide fuel cell and data points are simple where as case study II considers a 5 kW tubular fuel cell stack having 96 cells connected in series and the experimental I–V data points are highly non-linear at different temperatures and pressures. Firstly, the analysis is presented for the effect of number of data points on the evaluated objective function (SSE) and standard deviation of 30 SSE values for case study I. From Fig. 12a and Table 16 it is observed that with increase in number of data points, the obtained value of SSE and standard deviation from MFHA increases, which demonstrates that if the data of the model becomes larger, then the performance of MFHA is also affecting. Secondly, the analysis is presented based on the non-linearity of data set for case study II at different temperatures and pressures. From Table 16 and Fig. 12b, it can be seen that as the temperature of the SOFC system increases, the performance of MFHA is decreasing and thus SSE is increasing due to increased non-linearity in the data set. For different pressures, it is observed that, dataset for 1 atm, 2 atm and 5 atm is more non linear than data set at 3 atm and 4 atm. Thus, the performance of MFHA is poor at 1, 2 and 5 atm and thus SSE is increasing with increase in non-linearity in data set. However, it is important to note that the above discussions are limited to the application of MFHA on the SOFC model parameter estimation problem only. The analysis of developed MFHA on other complex optimization task is still a scope of research and should be further investigated in the future studies.

## Conclusions

This study proposes a modified fire hawk algorithm to evaluate unknown parameters of SOFC. The MFHA is well equipped with convergence speed and avoids local minima for identification of SOFC’s parameters. The algorithm’s effectiveness is first evaluated on 23 classical benchmark functions and the results are compared with original FHA. Results reveal that the MFHA algorithm performs better than FHA regarding indices like minimum value, maximum value, mean, and standard deviation. After benchmark evaluations, MFHA is adopted to identify seven parameters of SOFC for two different case studies. The main objective is to minimize the sum of squared error between experimental and estimated voltages. The robustness of MFHA in identifying the parameters is tested on two case studies viz a commercially available Siemens SOFC at four different temperatures and dynamic tabular 96 cell SOFC stack at different pressures and temperatures. Moreover, a comparative analysis for first case study is performed with other algorithms such as FHA, BES, AEO, GWO, WOA, MRFO, PSO, GWO-WOA, and HBA to evaluate the algorithm’s accuracy. A good coincidence of estimated polarization characteristics with experimental ones for both case studies validates the accuracy of MFHA and demonstrates that MFHA is a suitable identifier. Moreover, with least value of SSE for case study I as 1.04E−05, 2.30E−05, 1.03E−05, and 1.60E−05 at 1073 K, 1173 K, 1213 K, and 1273 K, respectively, confirms the accuracy of algorithm in evaluating seven optimal parameters of SOFC. Additionally, the reliability and robustness of MFHA are evaluated using statistical analysis, which concludes that with the least value of indices, including minimum, maximum, mean, and standard deviation of SSE, MFHA outperforms other algorithms. Convergence and box plot study further confirm the fast convergence speed and reliability of MFHA having the least median and small interquartile range. Furthermore, the non-parametric test is performed at different temperatures. It is concluded that MFHA outperforms other algorithms and obtains the first rank in solving SOFC model parameter identification problem. Furthermore, for case study II, the obtained values of SSE are 1.18E−03, 6.12E−03, 2.21E−02, 5.18E−02, and 6.00E−02 at temperatures of 873 K, 923 K, 973 K, 1023 K and 1073 K respectively, and 6.05E−02, 6.11E−02, 5.53E−02, 5.11E−02, and 6.64E−02 at pressures of 1 atm, 2 atm, 3 atm, 4 atm, and 5 atm respectively. Therefore, MFHA is proven to be an effective and robust approach in identifying SOFC model unknown parameters. The future work in this research will be related to integrating the derived accurate SOFC model into energy systems and investigate its performance under real scenarios. Moreover, the performance of developed MFHA is tested only on SOFC parameter identification, its investigation on other real world engineering application is still a scope of research.

## Data Availability

The datasets used and/or analysed during the current study available from the corresponding author on reasonable request.
